# A bioturbation classification of European marine infaunal invertebrates

**DOI:** 10.1002/ece3.769

**Published:** 2013-09-17

**Authors:** Ana M Queirós, Silvana N R Birchenough, Julie Bremner, Jasmin A Godbold, Ruth E Parker, Alicia Romero-Ramirez, Henning Reiss, Martin Solan, Paul J Somerfield, Carl Van Colen, Gert Van Hoey, Stephen Widdicombe

**Affiliations:** 1 Plymouth Marine LaboratoryProspect Place, The Hoe, Plymouth, PL1 3DH, U.K; 2 The Centre for Environment, Fisheries and Aquaculture SciencePakefield Road, Lowestoft, NR33 OHT, U.K; 3 Department of Ocean and Earth Science, National Oceanography Centre, University of SouthamptonWaterfront Campus, European Way, Southampton, SO14 3ZH, U.K; 4 EPOC – UMR5805, Université Bordeaux 1- CNRSStation Marine d'Arcachon, 2 Rue du Professeur Jolyet, Arcachon, 33120, France; 5 Faculty of Biosciences and Aquaculture, University of NordlandPostboks 1490, Bodø, 8049, Norway; 6 Department for Marine Research, Senckenberg Gesellschaft für NaturforschungSüdstrand 40, Wilhelmshaven, 26382, Germany; 7 Marine Biology Research Group, Ghent UniversityKrijgslaan 281/S8, Ghent, 9000, Belgium; 8 Bio-Environmental Research Group, Institute for Agriculture and Fisheries Research (ILVO-Fisheries)Ankerstraat 1, Ostend, 8400, Belgium

**Keywords:** Biodiversity, biogeochemical, ecosystem function, functional group, good environmental status, Marine Strategy Framework Directive, process, trait

## Abstract

Bioturbation, the biogenic modification of sediments through particle reworking and burrow ventilation, is a key mediator of many important geochemical processes in marine systems. In situ quantification of bioturbation can be achieved in a myriad of ways, requiring expert knowledge, technology, and resources not always available, and not feasible in some settings. Where dedicated research programmes do not exist, a practical alternative is the adoption of a trait-based approach to estimate community bioturbation potential (BP_c_). This index can be calculated from inventories of species, abundance and biomass data (routinely available for many systems), and a functional classification of organism traits associated with sediment mixing (less available). Presently, however, there is no agreed standard categorization for the reworking mode and mobility of benthic species. Based on information from the literature and expert opinion, we provide a functional classification for 1033 benthic invertebrate species from the northwest European continental shelf, as a tool to enable the standardized calculation of BP_c_ in the region. Future uses of this classification table will increase the comparability and utility of large-scale assessments of ecosystem processes and functioning influenced by bioturbation (e.g., to support legislation). The key strengths, assumptions, and limitations of BP_c_ as a metric are critically reviewed, offering guidelines for its calculation and application.

## Introduction

Marine soft-sediment habitats represent some of the most functionally important ecosystems on Earth, being characterized by a high biomass and diversity of invertebrate organisms that are fundamental to the mediation of a wealth of goods and services (Lotze et al. 2006; White et al. 2010; Widdicombe and Somerfield 2012). Infaunal invertebrates exhibit significant influence over benthic sedimentary geochemical environments in soft sediments through bioturbation, that is, the mixing of sediment and particulate materials carried out during foraging, feeding and burrow maintenance activities, and the enhancement of pore water and solute advection during burrow ventilation (Richter 1936; Rhoads 1974; Volkenborn et al. 2010). These processes influence oxygen, pH and redox gradients (Stahl et al. 2006; Pischedda et al. 2008; Queirós et al. 2011), metal cycling (Teal et al. 2009), sediment granulometry (Montserrat et al. 2009), pollutant release (Gilbert et al. 1994), macrofauna diversity (Volkenborn et al. 2007), bacterial activity and composition (Mermillod-Blondin and Rosenberg 2006; Gilbertson et al. 2012), and ultimately carbon (Kristensen 2001) and nitrogen cycling (Bertics et al. 2010). Hence, in light of anticipated changes to marine systems associated with human activity (Halpern et al. 2008; Hoegh-Guldberg and Bruno 2010), large-scale assessments of bioturbation can contribute to a better understanding of how of ecosystem functioning is mediated by biological activity.

**Figure 1:**
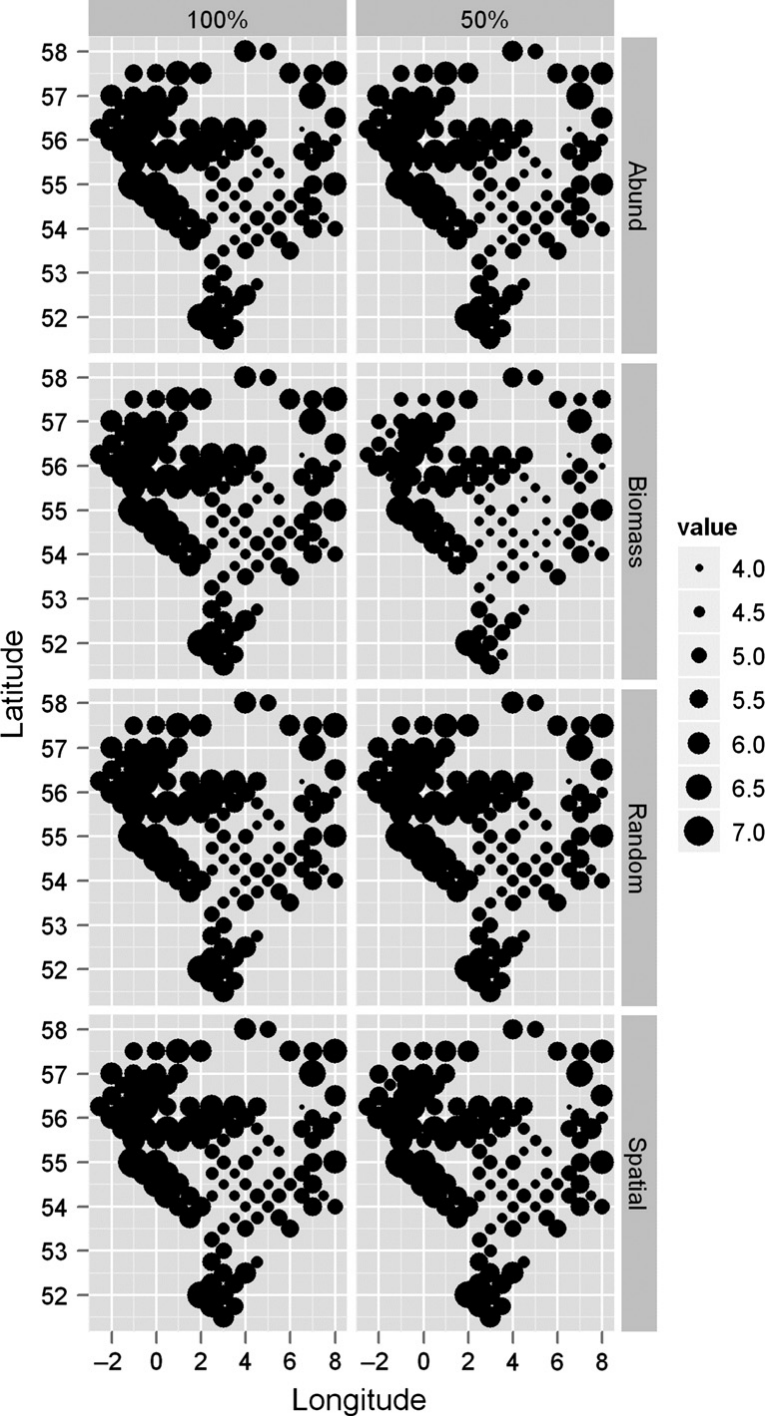
Percentage of sediment organic carbon (circles, diameter scaled to %) at each of 109 sites sampled during the North sea benthos Survey conducted by the Benthos Ecology Working Group of the International Council for the Exploration of the Sea in 1986 (left column, “100%”), and following the implementation of simulated trait based extinction scenarios (right column, “50%”). By combining measurements of ecosystem functions with information on relevant species traits (abundance, biomass, functional group and behaviour), empirically derived relationships between specific ecosystem functions and BP_c_ can be derived. In this case, the relationship between BP_c_ and sediment % of organic carbon. Species extinction scenarios can be simulated and the consequential changes in ecosystem functioning recalculated based on changes in community composition and/or structure following implementation of each scenario. In this example, the predicted levels of sediment organic carbon content are presented for a 50% reduction in species richness (right column, “50%”) following sequential local expiration of species ordered by the most abundant species within a site (top row), those with the largest biomass (second row from top), or by the most abundant species across the region (bottom row). The consequences for ecosystem functioning (here, sediment organic carbon content) following these ordered extinction scenarios contrast to a scenario where species are extirpated in a random order (third row from top). Implementing various trait-based extinction scenarios in this way provides insights on possible outcomes following, for example, changes in management or as a result of anthropogenic forcing. Modified from Solan et al. (2012).

Community bioturbation potential (BP_c_) is a metric first described by Solan et al. (2004a), which combines abundance and biomass data with information about the life traits of individual species or taxonomic groups. This information describes modes of sediment reworking (R_*i*_) and mobility (M_*i*_) of taxa in a dataset, two traits known to regulate biological sediment mixing, a key component of bioturbation (Solan 2000; and refereces therein; Solan et al. 2004b). BP_c_ is thus not a direct measure of the process of bioturbation. Rather, BP_c_ provides an estimate of the potential of a community to bioturbate. Hence, where macrofauna abundance and biomass data are available, BP_c_ provides a means to estimate the extent to which benthic communities are likely to affect important ecosystem properties that underpin ecosystem functioning. The consequences of environmentally driven changes in biodiversity to BP_c_, and its relation to ecosystem functioning, have been explored in this way in terrestrial (Bunker et al. 2005) and marine habitats (Solan et al. 2004a,b); at the local (Lohrer et al. 2010; Teal et al. 2013) and regional scales (Queirós et al. 2011; Birchenough et al. 2012; Solan et al. 2012); for different contexts (e.g., habitat structure and hypoxia, Queirós et al. 2011; Van Colen et al. 2012; Villnäs et al. 2012); and for a variety of ecosystem functions including productivity (Solan et al. 2012), nutrient cycling (Solan et al. 2004a), carbon storage (Bunker et al. 2005; Solan et al. 2012), and decomposition (Josefson et al. 2012). By calculating BP_c_ over time, or for different locations or scenarios, changes in the efficiency of the organism-sediment couple can be monitored for compliance in support of management and policy objectives (Painting et al. 2012; Van Hoey et al. 2013). For example, the effects of simulated changes in benthic community structure have previously been used to explore possible changes in ecosystem properties like sediment organic carbon at the North Sea scale, based on empirically derived relationships between BP_c_ and sediment organic carbon (Fig. 1). Similar uses of BP_c_ could invaluably contribute to an increased understanding of the role of ecosystem structure in the sustenance of marine functioning and its resilience to human activities, an urgent need under current European legislation (Marine Strategy Framework Directive, 2008/56/EC).

A significant obstacle in the widespread application of BP_c_, however, is the need for a standard classification scheme that is supported by the benthic research community. As a first step in fulfilling this research gap, we present the findings of the Study Group on Climate Related Benthic Processes in the North Sea, an expert group appointed by the International Council for the Exploration of the Sea (ICES SGCBNS). We present the conclusions of a series of dedicated workshops tasked with deriving a functional classification of northwest European marine invertebrate species to facilitate the calculation of BP_c_ in different regions of the North Atlantic.

## Methods

The classification of marine invertebrate infauna into bioturbation groups was carried out using 18 datasets compiled from northwest European waters (*n* = 1033 species). Following Swift (1993) and Solan et al. (2004a), each taxon (1) was scored on categorical scales that reflect increasing mobility (M_*i*_) from 1 (living in a fixed tube) to 4 (free three dimensional movement via burrow system), and increasing sediment reworking (R_*i*_) from 1 (epifauna that bioturbate at the sediment–water interface) to 5 (regenerators that excavate holes, transferring sediment at depth to the surface).


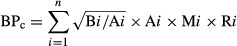


B_*i*_ and A_*i*_ are the biomass and abundance of species/taxon *i* in a sample. Trait scores were derived from an extensive review of published material and expert knowledge (consensus of 12 authors), and details of the scoring system are provided below. Species for which no published information was available were scored based on descriptions of species behavior and information on closely related species at the nearest taxonomic level. As BP_c_ captures information about sediment particle reworking, pelagic species and those living on hard substrates were not included. Sediment reworking functional types were also defined (Ft_*i*_), according to François et al. (1997), Solan (2000), and Gérino et al. (2003). Taxonomic information and Aphia ID (a unique species database identifier) were extracted from the World Register of Marine Species (2012).

## Results

Table 1 provides the classifications for mobility (M_*i*_) and sediment particle reworking (R_*i*_) assigned to the 1033 marine invertebrate species (and other taxa) from northwest European waters, and the associated sediment reworking functional types (Ft_*i*_). Please refer to the table for details of the scoring criteria.

**Table 1:** Bioturbation potential allocations for 1033 macrofaunal species. M_*i*_ and R_*i*_ are the reworking and mobility traits, and Ft_*i*_ is the corresponding sediment reworking functional types.

Scientific Name	Aphia ID	Ri	Mi	Fti	Phylum	Class	Order	Family
Grania	369702	4	3	B	Annelida	Clitellata	Enchytraeida	Caenogastropoda
*Tubificoides amplivasatus*	137570	4	3	B	Annelida	Clitellata	Haplotaxida	Tubificidae
*Tubificoides insularis*	137578	4	3	B	Annelida	Clitellata	Haplotaxida	Tubificidae
*Tubificoides pseudogaster*	137582	4	3	B	Annelida	Clitellata	Haplotaxida	Tubificidae
Oligochaeta	2036	4	3	B	Annelida	Clitellata		
*Cossura longocirrata*	129984	2	3	S	Annelida	Polychaeta		Cossuridae
*Chirimia biceps*	130277	3	2	UC/DC	Annelida	Polychaeta		Maldanidae
*Clymenura lankesteri*	130284	3	1	UC/DC	Annelida	Polychaeta		Maldanidae
Euclymene	129347	3	1	UC/DC	Annelida	Polychaeta		Maldanidae
Lumbriclymene	129350	3	1	UC/DC	Annelida	Polychaeta		Maldanidae
Nicomache	129357	3	1	UC/DC	Annelida	Polychaeta		Maldanidae
Rhodine	129363	3	1	UC	Annelida	Polychaeta		Maldanidae
*Ophelia borealis*	130491	4	3	B	Annelida	Polychaeta		Opheliidae
*Ophelina cylindricaudata*	465714	4	3	B	Annelida	Polychaeta		Opheliidae
*Ophelina modesta*	130507	4	3	B	Annelida	Polychaeta		Opheliidae
*Ophelina norvegica*	130508	4	3	B	Annelida	Polychaeta		Opheliidae
*Travisia forbesii*	130512	4	3	B	Annelida	Polychaeta		Opheliidae
*Orbinia norvegica*	156278	4	3	B	Annelida	Polychaeta		Orbiniidae
*Aricidea (Acmira) laubieri*	326587	2	3	S	Annelida	Polychaeta		Paraonidae
*Aricidea (Allia) roberti*	326598	2	3	S	Annelida	Polychaeta		Paraonidae
*Aricidea (Aricidea) wassi*	326605	2	3	S	Annelida	Polychaeta		Paraonidae
*Aricidea simonae*	130570	2	3	S	Annelida	Polychaeta		Paraonidae
*Aricidea suecica*	130572	2	3	S	Annelida	Polychaeta		Paraonidae
*Cirrophorus armatus*	152339	2	3	S	Annelida	Polychaeta		Paraonidae
*Paraonis fulgens*	146932	2	3	S	Annelida	Polychaeta		Paraonidae
Polygordius	129472	2	2	S	Annelida	Polychaeta		Polygordiidae
*Protodriloides chaetifer*	130837	2	2	S	Annelida	Polychaeta		Protodriloididae
*Polyphysia crassa*	130977	4	4	B	Annelida	Polychaeta		Scalibregmatidae
*Paramphinome jeffreysii*	129837	4	3	B	Annelida	Polychaeta	Amphinomida	Amphinomidae
*Pareurythoe borealis*	129838	4	3	B	Annelida	Polychaeta	Amphinomida	Amphinomidae
*Euphrosine foliosa*	130083	2	3	S	Annelida	Polychaeta	Amphinomida	Euphrosinidae
Capitellida	890	3	2	UC	Annelida	Polychaeta	Capitellida	
Ophryotrocha	129266	4	3	B	Annelida	Polychaeta	Eunicida	Dorvilleidae
*Parougia caeca*	130036	4	3	B	Annelida	Polychaeta	Eunicida	Dorvilleidae
*Parougia eliasoni*	130037	4	3	B	Annelida	Polychaeta	Eunicida	Dorvilleidae
*Parougia nigridentata*	130039	4	3	B	Annelida	Polychaeta	Eunicida	Dorvilleidae
*Protodorvillea kefersteini*	130041	4	3	B	Annelida	Polychaeta	Eunicida	Dorvilleidae
Schistomeringos	129274	4	3	B	Annelida	Polychaeta	Eunicida	Dorvilleidae
*Schistomeringos rudolphii*	154127	4	3	B	Annelida	Polychaeta	Eunicida	Dorvilleidae
Eunice	129278	4	3	B	Annelida	Polychaeta	Eunicida	Eunicidae
*Eunice dubitata*	130055	4	3	B	Annelida	Polychaeta	Eunicida	Eunicidae
*Eunice pennata*	130060	4	3	B	Annelida	Polychaeta	Eunicida	Eunicidae
Eunicidae	966	4	3	B	Annelida	Polychaeta	Eunicida	Eunicidae
*Lysidice ninetta*	130071	4	3	B	Annelida	Polychaeta	Eunicida	Eunicidae
Marphysa	129281	4	3	B	Annelida	Polychaeta	Eunicida	Eunicidae
*Marphysa bellii*	130072	4	3	B	Annelida	Polychaeta	Eunicida	Eunicidae
*Nematonereis unicornis*	594957	4	3	B	Annelida	Polychaeta	Eunicida	Eunicidae
Abyssoninoe	129331	4	3	B	Annelida	Polychaeta	Eunicida	Lumbrineridae
*Abyssoninoe hibernica*	146469	4	3	B	Annelida	Polychaeta	Eunicida	Lumbrineridae
*Lumbrineriopsis paradoxa*	130235	4	3	B	Annelida	Polychaeta	Eunicida	Lumbrineridae
Lumbrineris	129337	4	3	B	Annelida	Polychaeta	Eunicida	Lumbrineridae
*Lumbrineris agastos*	130236	4	3	B	Annelida	Polychaeta	Eunicida	Lumbrineridae
*Lumbrineris aniara*	130238	4	3	B	Annelida	Polychaeta	Eunicida	Lumbrineridae
*Lumbrineris fragilis*	152285	4	3	B	Annelida	Polychaeta	Eunicida	Lumbrineridae
*Lumbrineris gracilis*	130244	4	3	B	Annelida	Polychaeta	Eunicida	Lumbrineridae
*Lumbrineris latreilli*	130248	4	3	B	Annelida	Polychaeta	Eunicida	Lumbrineridae
*Lumbrineris magnidentata*	155360	4	3	B	Annelida	Polychaeta	Eunicida	Lumbrineridae
*Ninoe armoricana*	130254	4	3	B	Annelida	Polychaeta	Eunicida	Lumbrineridae
Scoletoma	129340	4	3	B	Annelida	Polychaeta	Eunicida	Lumbrineridae
*Scoletoma impatiens*	130263	4	3	B	Annelida	Polychaeta	Eunicida	Lumbrineridae
Arabella	129199	4	3	B	Annelida	Polychaeta	Eunicida	Oenonidae
*Arabella iricolor*	129854	4	3	B	Annelida	Polychaeta	Eunicida	Oenonidae
Drilonereis	129200	4	3	B	Annelida	Polychaeta	Eunicida	Oenonidae
*Drilonereis filum*	129856	4	3	B	Annelida	Polychaeta	Eunicida	Oenonidae
*Hyalinoecia tubicola*	130464	2	3	S	Annelida	Polychaeta	Eunicida	Onuphidae
*Nothria conchylega*	130467	2	3	S	Annelida	Polychaeta	Eunicida	Onuphidae
*Nothria hyperborea*	181500	2	3	S	Annelida	Polychaeta	Eunicida	Onuphidae
Onuphis	129404	2	3	S	Annelida	Polychaeta	Eunicida	Onuphidae
*Paradiopatra /quadricuspis*	130480	2	3	S	Annelida	Polychaeta	Eunicida	Onuphidae
Eunicida	895	4	3	B	Annelida	Polychaeta	Eunicida	
*Eupanthalis kinbergi*	129735	4	3	B	Annelida	Polychaeta	Phyllodocida	Acoetidae
*Aphrodita aculeata*	129840	4	3	B	Annelida	Polychaeta	Phyllodocida	Aphroditidae
Aphroditidae	938	4	3	B	Annelida	Polychaeta	Phyllodocida	Aphroditidae
Glycera	129296	4	3	B	Annelida	Polychaeta	Phyllodocida	Glyceridae
*Glycera alba*	130116	4	3	B	Annelida	Polychaeta	Phyllodocida	Glyceridae
*Glycera celtica*	130119	4	3	B	Annelida	Polychaeta	Phyllodocida	Glyceridae
*Glycera fallax*	336908	4	3	B	Annelida	Polychaeta	Phyllodocida	Glyceridae
*Glycera gigantea*	130122	4	3	B	Annelida	Polychaeta	Phyllodocida	Glyceridae
*Glycera lapidum*	130123	4	3	B	Annelida	Polychaeta	Phyllodocida	Glyceridae
*Glycera oxycephala*	130126	4	3	B	Annelida	Polychaeta	Phyllodocida	Glyceridae
*Glycera rouxii*	130127	4	3	B	Annelida	Polychaeta	Phyllodocida	Glyceridae
*Glycera tesselata*	130128	4	3	B	Annelida	Polychaeta	Phyllodocida	Glyceridae
*Glycera tridactyla*	130130	4	3	B	Annelida	Polychaeta	Phyllodocida	Glyceridae
*Glycera unicornis*	130131	4	3	B	Annelida	Polychaeta	Phyllodocida	Glyceridae
Glyceridae	952	4	3	B	Annelida	Polychaeta	Phyllodocida	Glyceridae
*Glycinde nordmanni*	130136	4	3	B	Annelida	Polychaeta	Phyllodocida	Goniadidae
Goniada	129300	4	3	B	Annelida	Polychaeta	Phyllodocida	Goniadidae
*Goniada maculata*	130140	4	3	B	Annelida	Polychaeta	Phyllodocida	Goniadidae
Goniadella	129301	4	3	B	Annelida	Polychaeta	Phyllodocida	Goniadidae
*Goniadella gracilis*	130145	4	3	B	Annelida	Polychaeta	Phyllodocida	Goniadidae
Gyptis	129307	4	3	B	Annelida	Polychaeta	Phyllodocida	Hesionidae
Hesionidae	946	4	3	B	Annelida	Polychaeta	Phyllodocida	Hesionidae
*Kefersteinia cirrata*	130164	4	3	B	Annelida	Polychaeta	Phyllodocida	Hesionidae
Microphthalmus	129313	2	3	S	Annelida	Polychaeta	Phyllodocida	Hesionidae
*Microphthalmus similis*	130176	2	3	S	Annelida	Polychaeta	Phyllodocida	Hesionidae
*Nereimyra punctata*	130185	4	3	B	Annelida	Polychaeta	Phyllodocida	Hesionidae
*Oxydromus agilis*	710683	4	3	B	Annelida	Polychaeta	Phyllodocida	Hesionidae
*Oxydromus flexuosus*	710680	4	3	B	Annelida	Polychaeta	Phyllodocida	Hesionidae
*Podarkeopsis capensis*	130195	4	3	B	Annelida	Polychaeta	Phyllodocida	Hesionidae
*Psamathe fusca*	152249	4	3	B	Annelida	Polychaeta	Phyllodocida	Hesionidae
*Syllidia armata*	130198	4	3	B	Annelida	Polychaeta	Phyllodocida	Hesionidae
*Aglaophamus agilis*	130343	4	3	B	Annelida	Polychaeta	Phyllodocida	Nephtyidae
Nephtyidae	956	4	3	B	Annelida	Polychaeta	Phyllodocida	Nephtyidae
Nephtys	129370	4	3	B	Annelida	Polychaeta	Phyllodocida	Nephtyidae
*Nephtys assimilis*	130353	4	3	B	Annelida	Polychaeta	Phyllodocida	Nephtyidae
*Nephtys caeca*	130355	4	3	B	Annelida	Polychaeta	Phyllodocida	Nephtyidae
*Nephtys cirrosa*	130357	4	3	B	Annelida	Polychaeta	Phyllodocida	Nephtyidae
*Nephtys hombergii*	130359	4	3	B	Annelida	Polychaeta	Phyllodocida	Nephtyidae
*Nephtys hystricis*	130360	4	3	B	Annelida	Polychaeta	Phyllodocida	Nephtyidae
*Nephtys incisa*	130362	4	3	B	Annelida	Polychaeta	Phyllodocida	Nephtyidae
*Nephtys kersivalensis*	130363	4	3	B	Annelida	Polychaeta	Phyllodocida	Nephtyidae
*Nephtys longosetosa*	130364	4	3	B	Annelida	Polychaeta	Phyllodocida	Nephtyidae
*Nephtys paradoxa*	130365	4	3	B	Annelida	Polychaeta	Phyllodocida	Nephtyidae
Nereididae	22496	4	4	B	Annelida	Polychaeta	Phyllodocida	Nereidae
*Alitta succinea*	234850	4	4	B	Annelida	Polychaeta	Phyllodocida	Nereididae
*Ceratocephale loveni*	130367	4	3	B	Annelida	Polychaeta	Phyllodocida	Nereididae
*Eunereis elittoralis*	130374	4	4	B	Annelida	Polychaeta	Phyllodocida	Nereididae
*Eunereis longissima*	130375	4	4	B	Annelida	Polychaeta	Phyllodocida	Nereididae
*Hediste diversicolor*	152302	4	4	B	Annelida	Polychaeta	Phyllodocida	Nereididae
*Micronereis variegata*	130380	4	4	B	Annelida	Polychaeta	Phyllodocida	Nereididae
*Neanthes fucata*	130387	4	4	B	Annelida	Polychaeta	Phyllodocida	Nereididae
*Neanthes irrorata*	130389	4	4	B	Annelida	Polychaeta	Phyllodocida	Nereididae
Nereis	129379	4	4	B	Annelida	Polychaeta	Phyllodocida	Nereididae
*Nereis pelagica*	130404	4	4	B	Annelida	Polychaeta	Phyllodocida	Nereididae
Nereis zonata	130407	4	4	B	Annelida	Polychaeta	Phyllodocida	Nereididae
*Websterinereis glauca*	130426	4	4	B	Annelida	Polychaeta	Phyllodocida	Nereididae
*Paralacydonia paradoxa*	130545	2	2	S	Annelida	Polychaeta	Phyllodocida	Paralacydoniidae
Pholoe	129439	2	2	S	Annelida	Polychaeta	Phyllodocida	Pholoidae
*Pholoe assimilis*	130598	2	2	S	Annelida	Polychaeta	Phyllodocida	Pholoidae
*Pholoe baltica*	130599	2	2	S	Annelida	Polychaeta	Phyllodocida	Pholoidae
*Pholoe inornata*	130601	2	2	S	Annelida	Polychaeta	Phyllodocida	Pholoidae
*Pholoe minuta*	130603	2	2	S	Annelida	Polychaeta	Phyllodocida	Pholoidae
*Pholoe pallida*	130604	2	2	S	Annelida	Polychaeta	Phyllodocida	Pholoidae
*Chaetoparia nilssoni*	130610	4	3	B	Annelida	Polychaeta	Phyllodocida	Phyllodocidae
Eteone	129443	4	3	B	Annelida	Polychaeta	Phyllodocida	Phyllodocidae
*Eteone barbata*	231870	4	3	B	Annelida	Polychaeta	Phyllodocida	Phyllodocidae
*Eteone flava*	130613	4	3	B	Annelida	Polychaeta	Phyllodocida	Phyllodocidae
*Eteone foliosa*	130614	4	3	B	Annelida	Polychaeta	Phyllodocida	Phyllodocidae
*Eteone longa*	130616	4	3	B	Annelida	Polychaeta	Phyllodocida	Phyllodocidae
Eulalia	129445	4	3	B	Annelida	Polychaeta	Phyllodocida	Phyllodocidae
*Eulalia mustela*	130631	4	3	B	Annelida	Polychaeta	Phyllodocida	Phyllodocidae
*Eulalia viridis*	130639	4	3	B	Annelida	Polychaeta	Phyllodocida	Phyllodocidae
Eumida	129446	4	3	B	Annelida	Polychaeta	Phyllodocida	Phyllodocidae
*Eumida bahusiensis*	130641	4	3	B	Annelida	Polychaeta	Phyllodocida	Phyllodocidae
*Eumida sanguinea*	130644	4	3	B	Annelida	Polychaeta	Phyllodocida	Phyllodocidae
*Hesionura elongata*	130649	4	3	B	Annelida	Polychaeta	Phyllodocida	Phyllodocidae
*Hypereteone foliosa*	152250	4	3	B	Annelida	Polychaeta	Phyllodocida	Phyllodocidae
*Mysta picta*	147026	4	3	B	Annelida	Polychaeta	Phyllodocida	Phyllodocidae
*Nereiphylla rubiginosa*	130659	4	3	B	Annelida	Polychaeta	Phyllodocida	Phyllodocidae
*Paranaitis kosteriensis*	130662	4	3	B	Annelida	Polychaeta	Phyllodocida	Phyllodocidae
Phyllodoce	129455	4	3	B	Annelida	Polychaeta	Phyllodocida	Phyllodocidae
*Phyllodoce (Anaitides) groenlandica*	130668	4	3	B	Annelida	Polychaeta	Phyllodocida	Phyllodocidae
*Phyllodoce groenlandica*	334506	4	3	B	Annelida	Polychaeta	Phyllodocida	Phyllodocidae
*Phyllodoce laminosa*	130670	4	3	B	Annelida	Polychaeta	Phyllodocida	Phyllodocidae
*Phyllodoce lineata*	334508	4	3	B	Annelida	Polychaeta	Phyllodocida	Phyllodocidae
*Phyllodoce longipes*	130673	4	3	B	Annelida	Polychaeta	Phyllodocida	Phyllodocidae
*Phyllodoce maculata*	334510	4	3	B	Annelida	Polychaeta	Phyllodocida	Phyllodocidae
*Phyllodoce mucosa*	334512	4	3	B	Annelida	Polychaeta	Phyllodocida	Phyllodocidae
*Phyllodoce rosea*	334514	4	3	B	Annelida	Polychaeta	Phyllodocida	Phyllodocidae
Phyllodocidae	931	4	3	B	Annelida	Polychaeta	Phyllodocida	Phyllodocidae
*Pirakia punctifera*	147104	4	3	B	Annelida	Polychaeta	Phyllodocida	Phyllodocidae
*Pseudomystides limbata*	130683	4	3	B	Annelida	Polychaeta	Phyllodocida	Phyllodocidae
*Sige fusigera*	130690	4	3	B	Annelida	Polychaeta	Phyllodocida	Phyllodocidae
*Ancistargis hamata*	130692	4	3	B	Annelida	Polychaeta	Phyllodocida	Pilargidae
*Ancistrosyllis groenlandica*	130695	4	3	B	Annelida	Polychaeta	Phyllodocida	Pilargidae
*Glyphohesione klatti*	130696	4	3	B	Annelida	Polychaeta	Phyllodocida	Pilargidae
*Pilargis verrucosa*	130700	4	3	B	Annelida	Polychaeta	Phyllodocida	Pilargidae
*Sigambra parva*	130702	4	3	B	Annelida	Polychaeta	Phyllodocida	Pilargidae
*Pisione remota*	130707	4	3	B	Annelida	Polychaeta	Phyllodocida	Pisionidae
*Acholoe squamosa*	146474	4	3	B	Annelida	Polychaeta	Phyllodocida	Polynoidae
*Alentia gelatinosa*	130722	4	3	B	Annelida	Polychaeta	Phyllodocida	Polynoidae
*Enipo elisabethae*	130737	4	3	B	Annelida	Polychaeta	Phyllodocida	Polynoidae
*Enipo kinbergi*	130738	4	3	B	Annelida	Polychaeta	Phyllodocida	Polynoidae
*Enipo kinbergi*	130738	4	3	B	Annelida	Polychaeta	Phyllodocida	Polynoidae
*Eunoe nodosa*	130745	4	3	B	Annelida	Polychaeta	Phyllodocida	Polynoidae
*Gattyana amondseni*	130748	4	3	B	Annelida	Polychaeta	Phyllodocida	Polynoidae
*Gattyana cirrhosa*	130749	4	3	B	Annelida	Polychaeta	Phyllodocida	Polynoidae
Harmothoe	129491	4	3	B	Annelida	Polychaeta	Phyllodocida	Polynoidae
*Harmothoe extenuata*	130762	4	3	B	Annelida	Polychaeta	Phyllodocida	Polynoidae
*Harmothoe fraserthomsoni*	130764	4	3	B	Annelida	Polychaeta	Phyllodocida	Polynoidae
*Harmothoe glabra*	571832	4	3	B	Annelida	Polychaeta	Phyllodocida	Polynoidae
*Harmothoe impar*	130770	4	3	B	Annelida	Polychaeta	Phyllodocida	Polynoidae
*Lepidonotus squamatus*	130801	1	1	E	Annelida	Polychaeta	Phyllodocida	Polynoidae
Malmgrenia	147006	4	3	B	Annelida	Polychaeta	Phyllodocida	Polynoidae
*Malmgrenia andreapolis*	147008	4	3	B	Annelida	Polychaeta	Phyllodocida	Polynoidae
Malmgreniella	129499	4	3	B	Annelida	Polychaeta	Phyllodocida	Polynoidae
*Malmgreniella arenicolae*	130810	4	3	B	Annelida	Polychaeta	Phyllodocida	Polynoidae
*Malmgreniella castanea*	130811	4	3	B	Annelida	Polychaeta	Phyllodocida	Polynoidae
*Malmgreniella darbouxi*	130812	4	3	B	Annelida	Polychaeta	Phyllodocida	Polynoidae
*Malmgreniella lilianae*	130814	4	3	B	Annelida	Polychaeta	Phyllodocida	Polynoidae
*Malmgreniella ljungmani*	130815	4	3	B	Annelida	Polychaeta	Phyllodocida	Polynoidae
*Malmgreniella lunulata*	130816	4	3	B	Annelida	Polychaeta	Phyllodocida	Polynoidae
*Malmgreniella mcintoshi*	130818	4	3	B	Annelida	Polychaeta	Phyllodocida	Polynoidae
*Malmgreniella polypapillata*	236712	4	3	B	Annelida	Polychaeta	Phyllodocida	Polynoidae
Polynoidae	939	4	3	B	Annelida	Polychaeta	Phyllodocida	Polynoidae
Polynoinae	155091	4	3	B	Annelida	Polychaeta	Phyllodocida	Polynoidae
*Labioleanira yhleni*	131067	4	3	B	Annelida	Polychaeta	Phyllodocida	Sigalionidae
*Neoleanira tetragona*	131069	4	3	B	Annelida	Polychaeta	Phyllodocida	Sigalionidae
*Sigalion mathildae*	131072	4	3	B	Annelida	Polychaeta	Phyllodocida	Sigalionidae
Sigalionidae	943	4	3	B	Annelida	Polychaeta	Phyllodocida	Sigalionidae
Sthenelais	129595	4	3	B	Annelida	Polychaeta	Phyllodocida	Sigalionidae
*Sthenelais boa*	131074	4	3	B	Annelida	Polychaeta	Phyllodocida	Sigalionidae
*Sthenelais limicola*	131077	4	3	B	Annelida	Polychaeta	Phyllodocida	Sigalionidae
*Ephesiella abyssorum*	131081	4	3	B	Annelida	Polychaeta	Phyllodocida	Sphaerodoridae
*Sphaerodorum gracilis*	131100	4	3	B	Annelida	Polychaeta	Phyllodocida	Sphaerodoridae
*Erinaceusyllis erinaceus*	195953	2	3	S	Annelida	Polychaeta	Phyllodocida	Syllidae
*Eurysyllis tuberculata*	131288	4	3	B	Annelida	Polychaeta	Phyllodocida	Syllidae
*Eusyllis assimilis*	131289	4	3	B	Annelida	Polychaeta	Phyllodocida	Syllidae
*Eusyllis blomstrandi*	131290	4	3	B	Annelida	Polychaeta	Phyllodocida	Syllidae
*Exogone (Exogone) naidina*	131304	4	3	B	Annelida	Polychaeta	Phyllodocida	Syllidae
*Exogone (Exogone) verugera*	131307	4	3	B	Annelida	Polychaeta	Phyllodocida	Syllidae
*Exogone (Parexogone) hebes*	131302	4	3	B	Annelida	Polychaeta	Phyllodocida	Syllidae
Myrianida	129659	2	2	S	Annelida	Polychaeta	Phyllodocida	Syllidae
*Myrianida prolifera*	238200	2	2	S	Annelida	Polychaeta	Phyllodocida	Syllidae
*Odontosyllis ctenostoma*	131325	4	3	B	Annelida	Polychaeta	Phyllodocida	Syllidae
*Odontosyllis gibba*	131328	4	3	B	Annelida	Polychaeta	Phyllodocida	Syllidae
Pionosyllis	129669	4	3	B	Annelida	Polychaeta	Phyllodocida	Syllidae
Proceraea	129671	2	2	S	Annelida	Polychaeta	Phyllodocida	Syllidae
*Prosphaerosyllis tetralix*	195980	2	3	S	Annelida	Polychaeta	Phyllodocida	Syllidae
*Sphaerosyllis bulbosa*	131379	2	3	S	Annelida	Polychaeta	Phyllodocida	Syllidae
*Sphaerosyllis hystrix*	131388	2	3	S	Annelida	Polychaeta	Phyllodocida	Syllidae
*Sphaerosyllis taylori*	131394	2	3	S	Annelida	Polychaeta	Phyllodocida	Syllidae
*Streptodonta pterochaeta*	238207	4	3	B	Annelida	Polychaeta	Phyllodocida	Syllidae
*Streptosyllis websteri*	131402	2	3	S	Annelida	Polychaeta	Phyllodocida	Syllidae
Syllidae	948	2	3	S	Annelida	Polychaeta	Phyllodocida	Syllidae
*Syllides benedicti*	131405	2	3	S	Annelida	Polychaeta	Phyllodocida	Syllidae
Syllis	129680	2	3	S	Annelida	Polychaeta	Phyllodocida	Syllidae
*Syllis armillaris*	131415	2	3	S	Annelida	Polychaeta	Phyllodocida	Syllidae
*Syllis cornuta*	157583	4	3	B	Annelida	Polychaeta	Phyllodocida	Syllidae
*Syllis gracilis*	131435	2	3	S	Annelida	Polychaeta	Phyllodocida	Syllidae
*Syllis hyalina*	131436	2	3	S	Annelida	Polychaeta	Phyllodocida	Syllidae
*Syllis prolifera*	131452	2	3	S	Annelida	Polychaeta	Phyllodocida	Syllidae
*Trypanosyllis (Trypanosyllis) coeliaca*	335151	2	3	S	Annelida	Polychaeta	Phyllodocida	Syllidae
*Trypanosyllis zebra*	131467	2	3	S	Annelida	Polychaeta	Phyllodocida	Syllidae
*Tomopteris (Johnstonella) helgolandica*	334946	1	2	E	Annelida	Polychaeta	Phyllodocida	Tomopteridae
*Galathowenia oculata*	146950	2	1	S	Annelida	Polychaeta	Sabellida	Oweniidae
Myriochele	129426	2	1	S	Annelida	Polychaeta	Sabellida	Oweniidae
*Myriochele heeri*	130542	2	1	S	Annelida	Polychaeta	Sabellida	Oweniidae
*Owenia fusiformis*	130544	2	1	S	Annelida	Polychaeta	Sabellida	Oweniidae
Oweniidae	975	2	1	S	Annelida	Polychaeta	Sabellida	Oweniidae
*Sabellaria spinulosa*	130867	1	1	E	Annelida	Polychaeta	Sabellida	Sabellariidae
Sabellariidae	979	1	1	E	Annelida	Polychaeta	Sabellida	Sabellariidae
*Branchiomma bombyx*	130878	2	2	S	Annelida	Polychaeta	Sabellida	Sabellidae
Chone	129525	2	1	S	Annelida	Polychaeta	Sabellida	Sabellidae
*Chone acustica*	130885	2	1	S	Annelida	Polychaeta	Sabellida	Sabellidae
*Chone duneri*	130888	2	1	S	Annelida	Polychaeta	Sabellida	Sabellidae
Euchone	129528	2	1	S	Annelida	Polychaeta	Sabellida	Sabellidae
*Euchone rubrocincta*	130909	2	1	S	Annelida	Polychaeta	Sabellida	Sabellidae
*Euchone southerni*	130910	2	1	S	Annelida	Polychaeta	Sabellida	Sabellidae
*Jasmineira candela*	130919	2	1	S	Annelida	Polychaeta	Sabellida	Sabellidae
*Jasmineira caudata*	130920	2	1	S	Annelida	Polychaeta	Sabellida	Sabellidae
*Jasmineira elegans*	130921	2	1	S	Annelida	Polychaeta	Sabellida	Sabellidae
*Paradialychone filicaudata*	558743	2	1	S	Annelida	Polychaeta	Sabellida	Sabellidae
*Potamethus murrayi*	416508	2	1	S	Annelida	Polychaeta	Sabellida	Sabellidae
*Potamilla reniformis*	155389	2	1	S	Annelida	Polychaeta	Sabellida	Sabellidae
*Pseudopotamilla reniformis*	130963	2	1	S	Annelida	Polychaeta	Sabellida	Sabellidae
*Sabella pavonina*	130967	2	1	S	Annelida	Polychaeta	Sabellida	Sabellidae
Sabellidae	985	2	1	S	Annelida	Polychaeta	Sabellida	Sabellidae
Apistobranchus	129198	2	3	S	Annelida	Polychaeta	Spionida	Apistobranchidae
*Apistobranchus tenuis*	129850	2	3	S	Annelida	Polychaeta	Spionida	Apistobranchidae
*Apistobranchus tullbergi*	129851	2	3	S	Annelida	Polychaeta	Spionida	Apistobranchidae
Chaetopteridae	918	3	1	UC/DC	Annelida	Polychaeta	Spionida	Chaetopteridae
*Chaetopterus variopedatus*	129914	3	1	UC/DC	Annelida	Polychaeta	Spionida	Chaetopteridae
*Spiochaetopterus costarum*	129922	3	1	UC/DC	Annelida	Polychaeta	Spionida	Chaetopteridae
*Spiochaetopterus typicus*	129924	3	1	UC/DC	Annelida	Polychaeta	Spionida	Chaetopteridae
Magelona	129341	2	2	S	Annelida	Polychaeta	Spionida	Magelonidae
*Magelona alleni*	130266	2	1	S	Annelida	Polychaeta	Spionida	Magelonidae
*Magelona filiformis*	130268	2	2	S	Annelida	Polychaeta	Spionida	Magelonidae
*Magelona johnstoni*	130269	2	2	S	Annelida	Polychaeta	Spionida	Magelonidae
*Magelona minuta*	130270	2	2	S	Annelida	Polychaeta	Spionida	Magelonidae
*Magelona mirabilis*	130271	2	2	S	Annelida	Polychaeta	Spionida	Magelonidae
Magelonidae	914	2	2	S	Annelida	Polychaeta	Spionida	Magelonidae
Poecilochaetidae	916	2	2	S	Annelida	Polychaeta	Spionida	Poecilochaetidae
*Poecilochaetus serpens*	130711	2	2	S	Annelida	Polychaeta	Spionida	Poecilochaetidae
Aonides	129605	3	2	UC/DC	Annelida	Polychaeta	Spionida	Spionidae
*Aonides oxycephala*	131106	3	2	UC/DC	Annelida	Polychaeta	Spionida	Spionidae
*Aonides paucibranchiata*	131107	3	2	UC/DC	Annelida	Polychaeta	Spionida	Spionidae
*Aurospio banyulensis*	146532	3	2	UC/DC	Annelida	Polychaeta	Spionida	Spionidae
*Dipolydora caulleryi*	131116	4	3	B	Annelida	Polychaeta	Spionida	Spionidae
*Dipolydora coeca*	131117	3	1	UC/DC	Annelida	Polychaeta	Spionida	Spionidae
*Dipolydora socialis*	131124	4	3	B	Annelida	Polychaeta	Spionida	Spionidae
Laonice	129613	3	1	UC/DC	Annelida	Polychaeta	Spionida	Spionidae
*Laonice bahusiensis*	131127	3	1	UC/DC	Annelida	Polychaeta	Spionida	Spionidae
*Laonice cirrata*	131128	3	1	UC/DC	Annelida	Polychaeta	Spionida	Spionidae
*Laonice sarsi*	131129	3	1	UC/DC	Annelida	Polychaeta	Spionida	Spionidae
*Malacoceros fuliginosus*	131131	3	2	UC/DC	Annelida	Polychaeta	Spionida	Spionidae
*Microspio atlantica*	131137	3	2	UC/DC	Annelida	Polychaeta	Spionida	Spionidae
*Minuspio cirrifera*	152392	3	2	UC/DC	Annelida	Polychaeta	Spionida	Spionidae
*Paraprionospio pinnata*	131140	3	2	UC/DC	Annelida	Polychaeta	Spionida	Spionidae
*Paraspio decorata*	334397	3	2	UC/DC	Annelida	Polychaeta	Spionida	Spionidae
Polydora	129619	3	1	UC/DC	Annelida	Polychaeta	Spionida	Spionidae
*Polydora ciliata*	131141	3	1	UC/DC	Annelida	Polychaeta	Spionida	Spionidae
*Polydora cornuta*	131143	3	1	UC/DC	Annelida	Polychaeta	Spionida	Spionidae
Prionospio	129620	3	2	UC/DC	Annelida	Polychaeta	Spionida	Spionidae
*Prionospio caspersi*	131152	3	2	UC/DC	Annelida	Polychaeta	Spionida	Spionidae
*Prionospio cirrifera*	131153	3	2	UC/DC	Annelida	Polychaeta	Spionida	Spionidae
*Prionospio dubia*	131155	3	2	UC/DC	Annelida	Polychaeta	Spionida	Spionidae
*Prionospio ehlersi*	131156	3	2	UC/DC	Annelida	Polychaeta	Spionida	Spionidae
*Prionospio fallax*	131157	3	2	UC/DC	Annelida	Polychaeta	Spionida	Spionidae
*Prionospio malmgreni*	131159	3	2	UC/DC	Annelida	Polychaeta	Spionida	Spionidae
*Prionospio multibranchiata*	131160	3	2	UC/DC	Annelida	Polychaeta	Spionida	Spionidae
*Prionospio saldanha*	338540	3	2	UC/DC	Annelida	Polychaeta	Spionida	Spionidae
*Prionospio sexoculata*	131163	3	2	UC/DC	Annelida	Polychaeta	Spionida	Spionidae
*Prionospio steenstrupi*	131164	3	2	UC/DC	Annelida	Polychaeta	Spionida	Spionidae
*Pseudopolydora antennata*	131166	3	1	UC/DC	Annelida	Polychaeta	Spionida	Spionidae
*Pseudopolydora paucibranchiata*	131168	3	1	UC/DC	Annelida	Polychaeta	Spionida	Spionidae
*Pseudopolydora pulchra*	131169	3	1	UC/DC	Annelida	Polychaeta	Spionida	Spionidae
*Pygospio elegans*	131170	3	1	UC/DC	Annelida	Polychaeta	Spionida	Spionidae
Scolelepis	129623	3	2	UC/DC	Annelida	Polychaeta	Spionida	Spionidae
*Scolelepis (Scolelepis) foliosa*	334741	3	2	UC/DC	Annelida	Polychaeta	Spionida	Spionidae
*Scolelepis (Scolelepis) squamata*	157566	3	2	UC/DC	Annelida	Polychaeta	Spionida	Spionidae
*Scolelepis bonnieri*	131171	3	2	UC/DC	Annelida	Polychaeta	Spionida	Spionidae
*Scolelepis korsuni*	131174	3	2	UC/DC	Annelida	Polychaeta	Spionida	Spionidae
*Scolelepis tridentata*	131178	3	2	UC/DC	Annelida	Polychaeta	Spionida	Spionidae
Spio	129625	3	2	UC/DC	Annelida	Polychaeta	Spionida	Spionidae
*Spio armata*	131180	3	2	UC/DC	Annelida	Polychaeta	Spionida	Spionidae
*Spio filicornis*	131183	3	2	UC/DC	Annelida	Polychaeta	Spionida	Spionidae
*Spio goniocephala*	131184	3	2	UC/DC	Annelida	Polychaeta	Spionida	Spionidae
*Spio martinensis*	131185	3	2	UC/DC	Annelida	Polychaeta	Spionida	Spionidae
*Spio multioculata*	131186	3	2	UC/DC	Annelida	Polychaeta	Spionida	Spionidae
Spionidae	913	3	2	UC/DC	Annelida	Polychaeta	Spionida	Spionidae
Spiophanes	129626	3	1	UC/DC	Annelida	Polychaeta	Spionida	Spionidae
*Spiophanes bombyx*	131187	3	1	UC/DC	Annelida	Polychaeta	Spionida	Spionidae
*Spiophanes kroyeri*	131188	3	1	UC/DC	Annelida	Polychaeta	Spionida	Spionidae
*Spiophanes wigleyi*	131190	3	1	UC/DC	Annelida	Polychaeta	Spionida	Spionidae
*Streblospio benedicti*	131191	3	2	UC/DC	Annelida	Polychaeta	Spionida	Spionidae
*Macrochaeta clavicornis*	129745	2	2	S	Annelida	Polychaeta	Terebellida	Acrocirridae
*Macrochaeta helgolandica*	129746	2	2	S	Annelida	Polychaeta	Terebellida	Acrocirridae
Ampharete	129155	3	2	UC/DC	Annelida	Polychaeta	Terebellida	Ampharetidae
*Ampharete acutifrons*	129775	3	2	UC/DC	Annelida	Polychaeta	Terebellida	Ampharetidae
*Ampharete baltica*	129776	3	2	UC/DC	Annelida	Polychaeta	Terebellida	Ampharetidae
*Ampharete falcata*	129777	3	2	UC/DC	Annelida	Polychaeta	Terebellida	Ampharetidae
*Ampharete finmarchica*	129778	3	2	UC/DC	Annelida	Polychaeta	Terebellida	Ampharetidae
*Ampharete grubei*	152272	3	2	UC/DC	Annelida	Polychaeta	Terebellida	Ampharetidae
Ampharetidae	981	3	2	UC/DC	Annelida	Polychaeta	Terebellida	Ampharetidae
*Amphicteis gunneri*	129784	3	2	UC/DC	Annelida	Polychaeta	Terebellida	Ampharetidae
*Amphicteis midas*	129785	3	2	UC/DC	Annelida	Polychaeta	Terebellida	Ampharetidae
*Amythasides macroglossus*	129788	3	2	UC/DC	Annelida	Polychaeta	Terebellida	Ampharetidae
*Anobothrus gracilis*	129789	3	1	UC/DC	Annelida	Polychaeta	Terebellida	Ampharetidae
*Melinna cristata*	129804	3	1	UC/DC	Annelida	Polychaeta	Terebellida	Ampharetidae
*Melinna elisabethae*	129805	3	1	UC/DC	Annelida	Polychaeta	Terebellida	Ampharetidae
*Melinna palmata*	129808	3	1	UC/DC	Annelida	Polychaeta	Terebellida	Ampharetidae
*Mugga wahrbergi*	129813	3	2	UC/DC	Annelida	Polychaeta	Terebellida	Ampharetidae
*Pterolysippe vanelli*	334692	3	1	UC/DC	Annelida	Polychaeta	Terebellida	Ampharetidae
*Samytha sexcirrata*	129819	3	2	UC/DC	Annelida	Polychaeta	Terebellida	Ampharetidae
*Sosane sulcata*	129821	3	2	UC/DC	Annelida	Polychaeta	Terebellida	Ampharetidae
Aphelochaeta	129240	2	2	S	Annelida	Polychaeta	Terebellida	Cirratulidae
*Aphelochaeta filiformis*	129937	2	2	S	Annelida	Polychaeta	Terebellida	Cirratulidae
*Aphelochaeta marioni*	129938	2	2	S	Annelida	Polychaeta	Terebellida	Cirratulidae
Caulleriella	129241	2	2	S	Annelida	Polychaeta	Terebellida	Cirratulidae
*Caulleriella alata*	129943	2	2	S	Annelida	Polychaeta	Terebellida	Cirratulidae
*Caulleriella bioculata*	129944	2	2	S	Annelida	Polychaeta	Terebellida	Cirratulidae
*Caulleriella killariensis*	129945	2	2	S	Annelida	Polychaeta	Terebellida	Cirratulidae
*Caulleriella zetlandica*	129948	2	2	S	Annelida	Polychaeta	Terebellida	Cirratulidae
*Caulleriella zetlandica*	129948	2	2	S	Annelida	Polychaeta	Terebellida	Cirratulidae
Chaetozone	129242	2	2	S	Annelida	Polychaeta	Terebellida	Cirratulidae
*Chaetozone christiei*	152217	2	2	S	Annelida	Polychaeta	Terebellida	Cirratulidae
*Chaetozone gibber*	129953	2	2	S	Annelida	Polychaeta	Terebellida	Cirratulidae
*Chaetozone setosa*	129955	2	2	S	Annelida	Polychaeta	Terebellida	Cirratulidae
Cirratulidae	919	2	2	S	Annelida	Polychaeta	Terebellida	Cirratulidae
Cirratulus	129243	2	2	S	Annelida	Polychaeta	Terebellida	Cirratulidae
*Cirratulus caudatus*	129957	2	2	S	Annelida	Polychaeta	Terebellida	Cirratulidae
*Cirriformia tentaculata*	129964	2	2	S	Annelida	Polychaeta	Terebellida	Cirratulidae
*Monticellina dorsobranchialis*	129972	2	2	S	Annelida	Polychaeta	Terebellida	Cirratulidae
*Monticellina heterochaeta*	129973	2	2	S	Annelida	Polychaeta	Terebellida	Cirratulidae
Tharyx	129249	2	2	S	Annelida	Polychaeta	Terebellida	Cirratulidae
Fauveliopsis	129288	2	2	S	Annelida	Polychaeta	Terebellida	Fauveliopsidae
*Brada villosa*	130099	3	2	UC/DC	Annelida	Polychaeta	Terebellida	Flabelligeridae
*Diplocirrus glaucus*	130100	3	2	UC	Annelida	Polychaeta	Terebellida	Flabelligeridae
Flabelligeridae	976	3	2	UC	Annelida	Polychaeta	Terebellida	Flabelligeridae
*Pherusa monilifera*	130112	3	2	UC	Annelida	Polychaeta	Terebellida	Flabelligeridae
*Pherusa plumosa*	130113	3	2	UC	Annelida	Polychaeta	Terebellida	Flabelligeridae
*Amphictene auricoma*	152448	3	1	UC	Annelida	Polychaeta	Terebellida	Pectinariidae
*Lagis koreni*	152367	3	1	UC	Annelida	Polychaeta	Terebellida	Pectinariidae
Pectinaria	129437	3	1	UC	Annelida	Polychaeta	Terebellida	Pectinariidae
*Pectinaria (Amphictene) auricoma*	130590	3	1	UC	Annelida	Polychaeta	Terebellida	Pectinariidae
Pectinariidae	980	3	1	UC	Annelida	Polychaeta	Terebellida	Pectinariidae
*Sternaspis scutata*	131242	4	3	B	Annelida	Polychaeta	Terebellida	Sternaspidae
*Amaeana trilobata*	131471	3	1	UC/DC	Annelida	Polychaeta	Terebellida	Terebellidae
*Axionice maculata*	131484	3	1	DC	Annelida	Polychaeta	Terebellida	Terebellidae
*Eupolymnia nesidensis*	131490	1	1	E	Annelida	Polychaeta	Terebellida	Terebellidae
*Hauchiella tribullata*	152389	3	1	DC	Annelida	Polychaeta	Terebellida	Terebellidae
Lanice	129697	3	1	DC	Annelida	Polychaeta	Terebellida	Terebellidae
*Lanice conchilega*	131495	3	1	DC	Annelida	Polychaeta	Terebellida	Terebellidae
*Lysilla loveni*	131500	3	1	DC	Annelida	Polychaeta	Terebellida	Terebellidae
*Neoamphitrite affinis*	131502	3	1	DC	Annelida	Polychaeta	Terebellida	Terebellidae
*Nicolea zostericola*	131508	3	1	DC	Annelida	Polychaeta	Terebellida	Terebellidae
*Paramphitrite birulai*	152454	2	2	S	Annelida	Polychaeta	Terebellida	Terebellidae
*Phisidia aurea*	131513	3	1	DC	Annelida	Polychaeta	Terebellida	Terebellidae
*Pista cristata*	131516	3	1	DC	Annelida	Polychaeta	Terebellida	Terebellidae
*Pista lornensis*	154972	3	1	DC	Annelida	Polychaeta	Terebellida	Terebellidae
*Pistella lornensis*	131522	3	1	DC	Annelida	Polychaeta	Terebellida	Terebellidae
Polycirrus	129710	3	1	DC	Annelida	Polychaeta	Terebellida	Terebellidae
*Polycirrus medusa*	131531	3	1	DC	Annelida	Polychaeta	Terebellida	Terebellidae
*Streblosoma bairdi*	131538	3	1	DC	Annelida	Polychaeta	Terebellida	Terebellidae
*Streblosoma intestinale*	131540	3	1	DC	Annelida	Polychaeta	Terebellida	Terebellidae
Terebella	129713	3	1	DC	Annelida	Polychaeta	Terebellida	Terebellidae
Terebellidae	982	3	1	DC	Annelida	Polychaeta	Terebellida	Terebellidae
*Thelepus cincinnatus*	131543	3	1	DC	Annelida	Polychaeta	Terebellida	Terebellidae
*Thelepus setosus*	131544	3	1	DC	Annelida	Polychaeta	Terebellida	Terebellidae
*Terebellides stroemii*	131573	3	1	DC	Annelida	Polychaeta	Terebellida	Trichobranchidae
*Trichobranchidae*	983	3	1	DC	Annelida	Polychaeta	Terebellida	Trichobranchidae
*Trichobranchus glacialis*	131574	3	1	DC	Annelida	Polychaeta	Terebellida	Trichobranchidae
*Trichobranchus roseus*	131575	3	1	DC	Annelida	Polychaeta	Terebellida	Trichobranchidae
Arenicolides	129207	3	2	UC	Annelida	Polychaeta		Arenicolidae
Capitella	129211	3	2	UC	Annelida	Polychaeta		Capitellidae
*Capitella capitata*	129876	3	2	UC	Annelida	Polychaeta		Capitellidae
*Capitella minima*	129879	3	2	UC	Annelida	Polychaeta		Capitellidae
Capitellidae	921	3	2	UC	Annelida	Polychaeta		Capitellidae
*Heteromastus filiformis*	129884	3	2	UC	Annelida	Polychaeta		Capitellidae
*Mediomastus fragilis*	129892	3	2	UC	Annelida	Polychaeta		Capitellidae
Notomastus	129220	3	2	UC	Annelida	Polychaeta		Capitellidae
*Notomastus latericeus*	129898	3	2	UC	Annelida	Polychaeta		Capitellidae
*Peresiella clymenoides*	129906	3	2	UC	Annelida	Polychaeta		Capitellidae
*Pseudoleiocapitella*	129226	3	2	UC	Annelida	Polychaeta		Capitellidae
Cossuridae	908	2	3	S	Annelida	Polychaeta		Cossuridae
*Asychis biceps*	146523	3	1	UC	Annelida	Polychaeta		Maldanidae
*Clymenella torquata*	130279	3	1	UC	Annelida	Polychaeta		Maldanidae
Clymenura	129346	3	1	UC	Annelida	Polychaeta		Maldanidae
*Euclymene droebachiensis*	130291	3	1	UC	Annelida	Polychaeta		Maldanidae
*Euclymene lombricoides*	209899	3	1	UC	Annelida	Polychaeta		Maldanidae
*Euclymene oerstedi*	130294	3	1	UC	Annelida	Polychaeta		Maldanidae
*Heteroclymene robusta*	146978	3	1	UC	Annelida	Polychaeta		Maldanidae
*Johnstonia clymenoides*	130298	3	1	UC	Annelida	Polychaeta		Maldanidae
*Macroclymene santandarensis*	130301	3	1	UC	Annelida	Polychaeta		Maldanidae
Maldanidae	923	3	1	UC	Annelida	Polychaeta		Maldanidae
Notoproctus	129358	3	1	UC	Annelida	Polychaeta		Maldanidae
*Praxillella affinis*	130322	3	1	UC	Annelida	Polychaeta		Maldanidae
*Praxillella gracilis*	130324	3	1	UC	Annelida	Polychaeta		Maldanidae
*Rhodine gracilior*	130330	3	1	UC	Annelida	Polychaeta		Maldanidae
*Euzonus flabelligerus*	130487	4	3	B	Annelida	Polychaeta		Opheliidae
*Ophelia limacina*	130494	4	3	B	Annelida	Polychaeta		Opheliidae
Opheliidae	924	4	3	B	Annelida	Polychaeta		Opheliidae
*Ophelina acuminata*	130500	4	3	B	Annelida	Polychaeta		Opheliidae
*Polyophthalmus pictus*	130510	4	3	B	Annelida	Polychaeta		Opheliidae
*Orbinia (Orbinia) sertulata*	334310	4	3	B	Annelida	Polychaeta		Orbiniidae
Orbiniidae	902	4	3	B	Annelida	Polychaeta		Orbiniidae
Scoloplos	129425	4	3	B	Annelida	Polychaeta		Orbiniidae
*Scoloplos (Scoloplos) armiger*	334772	4	3	B	Annelida	Polychaeta		Orbiniidae
*Aedicira mediterranea*	130546	2	3	S	Annelida	Polychaeta		Paraonidae
Aricidea	129430	2	3	S	Annelida	Polychaeta		Paraonidae
*Aricidea (Acmira) catherinae*	333034	2	3	S	Annelida	Polychaeta		Paraonidae
*Aricidea (Acmira) cerrutii*	525497	2	3	S	Annelida	Polychaeta		Paraonidae
*Aricidea (Acmira) lopezi*	333036	2	3	S	Annelida	Polychaeta		Paraonidae
*Aricidea minuta*	130564	2	3	S	Annelida	Polychaeta		Paraonidae
*Cirrophorus branchiatus*	130576	2	3	S	Annelida	Polychaeta		Paraonidae
*Levinsenia gracilis*	130578	2	3	S	Annelida	Polychaeta		Paraonidae
*Paradoneis lyra*	130585	2	3	S	Annelida	Polychaeta		Paraonidae
Paraonidae	903	2	3	S	Annelida	Polychaeta		Paraonidae
*Polygordius appendiculatus*	130712	2	2	S	Annelida	Polychaeta		Polygordiidae
*Polygordius lacteus*	130714	2	2	S	Annelida	Polychaeta		Polygordiidae
Protodriloides	129513	2	2	S	Annelida	Polychaeta		Protodriloididae
*Asclerocheilus intermedius*	130974	4	4	B	Annelida	Polychaeta		Scalibregmatidae
*Scalibregma celticum*	130979	4	4	B	Annelida	Polychaeta		Scalibregmatidae
*Scalibregma inflatum*	130980	4	4	B	Annelida	Polychaeta		Scalibregmatidae
Scalibregmatidae	925	4	4	B	Annelida	Polychaeta		Scalibregmatidae
Acidostoma	101586	2	3	S	Arthropoda	Malacostraca	Amphipoda	Acidostomatidae
Ampelisca	101445	2	1	S	Arthropoda	Malacostraca	Amphipoda	Ampeliscidae
*Ampelisca armoricana*	101888	2	1	S	Arthropoda	Malacostraca	Amphipoda	Ampeliscidae
*Ampelisca brevicornis*	101891	2	1	S	Arthropoda	Malacostraca	Amphipoda	Ampeliscidae
*Ampelisca diadema*	101896	2	1	S	Arthropoda	Malacostraca	Amphipoda	Ampeliscidae
*Ampelisca gibba*	101898	2	1	S	Arthropoda	Malacostraca	Amphipoda	Ampeliscidae
*Ampelisca macrocephala*	101908	2	1	S	Arthropoda	Malacostraca	Amphipoda	Ampeliscidae
*Ampelisca sarsi*	101923	2	1	S	Arthropoda	Malacostraca	Amphipoda	Ampeliscidae
*Ampelisca spinipes*	101928	2	1	S	Arthropoda	Malacostraca	Amphipoda	Ampeliscidae
*Ampelisca tenuicornis*	101930	2	1	S	Arthropoda	Malacostraca	Amphipoda	Ampeliscidae
*Ampelisca typica*	101933	2	1	S	Arthropoda	Malacostraca	Amphipoda	Ampeliscidae
*Ampelisca vervecei*	101937	2	1	S	Arthropoda	Malacostraca	Amphipoda	Ampeliscidae
Byblis	101446	2	4	S	Arthropoda	Malacostraca	Amphipoda	Ampeliscidae
*Haploops tubicola*	101958	2	1	S	Arthropoda	Malacostraca	Amphipoda	Ampeliscidae
*Amphilochoides boecki*	101960	2	3	B	Arthropoda	Malacostraca	Amphipoda	Amphilochidae
*Amphilochoides serratipes*	101963	2	3	B	Arthropoda	Malacostraca	Amphipoda	Amphilochidae
*Amphilochus manudens*	101967	2	3	B	Arthropoda	Malacostraca	Amphipoda	Amphilochidae
*Amphilochus neapolitanus*	101968	2	3	B	Arthropoda	Malacostraca	Amphipoda	Amphilochidae
*Gitana sarsi*	101977	2	3	B	Arthropoda	Malacostraca	Amphipoda	Amphilochidae
*Paramphilochoides odontonyx*	101982	2	3	B	Arthropoda	Malacostraca	Amphipoda	Amphilochidae
*Ampithoe ramondi*	102000	1	1	E	Arthropoda	Malacostraca	Amphipoda	Ampithoidae
*Aora gracilis*	102012	1	3	S	Arthropoda	Malacostraca	Amphipoda	Aoridae
*Aora typica*	146895	1	3	S	Arthropoda	Malacostraca	Amphipoda	Aoridae
Aoridae	101368	1	3	S	Arthropoda	Malacostraca	Amphipoda	Aoridae
*Autonoe longipes*	102021	1	3	S	Arthropoda	Malacostraca	Amphipoda	Aoridae
Microdeutopus	101471	1	3	S	Arthropoda	Malacostraca	Amphipoda	Aoridae
*Argissa hamatipes*	102064	2	3	S	Arthropoda	Malacostraca	Amphipoda	Argissidae
Atylus	101497	2	3	S	Arthropoda	Malacostraca	Amphipoda	Atylidae
*Atylus guttatus*	102127	2	3	S	Arthropoda	Malacostraca	Amphipoda	Atylidae
*Atylus swammerdami*	102131	2	3	S	Arthropoda	Malacostraca	Amphipoda	Atylidae
*Atylus vedlomensis*	102132	2	3	S	Arthropoda	Malacostraca	Amphipoda	Atylidae
*Nototropis falcatus*	102139	2	3	S	Arthropoda	Malacostraca	Amphipoda	Atylidae
Apherusa	101509	2	3	S	Arthropoda	Malacostraca	Amphipoda	Calliopiidae
*Apherusa bispinosa*	102160	2	3	S	Arthropoda	Malacostraca	Amphipoda	Calliopiidae
*Apherusa clevei*	102164	2	3	S	Arthropoda	Malacostraca	Amphipoda	Calliopiidae
*Apherusa jurinei*	102168	2	3	S	Arthropoda	Malacostraca	Amphipoda	Calliopiidae
*Apherusa ovalipes*	102172	2	3	S	Arthropoda	Malacostraca	Amphipoda	Calliopiidae
*Caprella acanthifera*	101822	2	2	S	Arthropoda	Malacostraca	Amphipoda	Caprellidae
*Caprella linearis*	101839	2	2	S	Arthropoda	Malacostraca	Amphipoda	Caprellidae
*Pariambus typicus*	101857	2	2	S	Arthropoda	Malacostraca	Amphipoda	Caprellidae
*Parvipalpus capillaceus*	101858	2	2	S	Arthropoda	Malacostraca	Amphipoda	Caprellidae
*Phtisica marina*	101864	2	2	S	Arthropoda	Malacostraca	Amphipoda	Caprellidae
*Pseudoprotella phasma*	101871	2	2	S	Arthropoda	Malacostraca	Amphipoda	Caprellidae
Cheirocratus	101669	2	3	S	Arthropoda	Malacostraca	Amphipoda	Cheirocratidae
*Cheirocratus intermedius*	102795	2	3	S	Arthropoda	Malacostraca	Amphipoda	Cheirocratidae
*Cheirocratus sundevalli*	102798	2	3	S	Arthropoda	Malacostraca	Amphipoda	Cheirocratidae
*Apocorophium lacustre*	148594	2	4	S	Arthropoda	Malacostraca	Amphipoda	Corophiidae
Corophium	101489	2	4	S	Arthropoda	Malacostraca	Amphipoda	Corophiidae
*Crassicorophium bonellii*	237004	2	4	S	Arthropoda	Malacostraca	Amphipoda	Corophiidae
Leptocheirus	101470	2	4	S	Arthropoda	Malacostraca	Amphipoda	Corophiidae
*Leptocheirus hirsutimanus*	102036	2	4	S	Arthropoda	Malacostraca	Amphipoda	Corophiidae
*Leptocheirus mariae*	102038	2	4	S	Arthropoda	Malacostraca	Amphipoda	Corophiidae
*Leptocheirus pectinatus*	102039	2	4	S	Arthropoda	Malacostraca	Amphipoda	Corophiidae
*Leptocheirus tricristatus*	102041	2	4	S	Arthropoda	Malacostraca	Amphipoda	Corophiidae
*Medicorophium affine*	423507	2	4	S	Arthropoda	Malacostraca	Amphipoda	Corophiidae
*Monocorophium acherusicum*	225814	2	4	S	Arthropoda	Malacostraca	Amphipoda	Corophiidae
*Monocorophium insidiosum*	148592	2	4	S	Arthropoda	Malacostraca	Amphipoda	Corophiidae
*Monocorophium sextonae*	148603	2	4	S	Arthropoda	Malacostraca	Amphipoda	Corophiidae
Protomedeia	101574	2	4	S	Arthropoda	Malacostraca	Amphipoda	Corophiidae
*Peltocoxa brevirostris*	101983	2	3	S	Arthropoda	Malacostraca	Amphipoda	Cyproideidae
Dexamine	101498	2	3	S	Arthropoda	Malacostraca	Amphipoda	Dexaminidae
*Dexamine spinosa*	102135	2	3	S	Arthropoda	Malacostraca	Amphipoda	Dexaminidae
*Guernea (Guernea) coalita*	102137	2	3	S	Arthropoda	Malacostraca	Amphipoda	Dexaminidae
*Tritaeta gibbosa*	102141	2	3	S	Arthropoda	Malacostraca	Amphipoda	Dexaminidae
Dyopedos	101736	2	3	S	Arthropoda	Malacostraca	Amphipoda	Dulichiidae
*Dyopedos monacantha*	103042	2	3	S	Arthropoda	Malacostraca	Amphipoda	Dulichiidae
*Eusirus longipes*	102202	2	3	S	Arthropoda	Malacostraca	Amphipoda	Eusiridae
*Haustorius arenarius*	102317	2	3	S	Arthropoda	Malacostraca	Amphipoda	Haustoriidae
*Hyperia galba*	103251	2	3	S	Arthropoda	Malacostraca	Amphipoda	Hyperiidae
Iphimedia	101554	2	3	S	Arthropoda	Malacostraca	Amphipoda	Iphimediidae
*Iphimedia minuta*	102345	2	3	S	Arthropoda	Malacostraca	Amphipoda	Iphimediidae
*Iphimedia obesa*	102347	2	3	S	Arthropoda	Malacostraca	Amphipoda	Iphimediidae
*Ericthonius punctatus*	102408	2	1	S	Arthropoda	Malacostraca	Amphipoda	Ischyroceridae
*Jassa falcata*	102431	1	1	E	Arthropoda	Malacostraca	Amphipoda	Ischyroceridae
*Siphonoecetes (Centraloecetes) kroyeranus*	102111	2	1	S	Arthropoda	Malacostraca	Amphipoda	Ischyroceridae
*Siphonoecetes (Centraloecetes) neapolitanus*	102112	2	1	S	Arthropoda	Malacostraca	Amphipoda	Ischyroceridae
*Siphonoecetes (Centraloecetes) striatus*	102115	1	1	S	Arthropoda	Malacostraca	Amphipoda	Ischyroceridae
*Leucothoe incisa*	102460	2	3	S	Arthropoda	Malacostraca	Amphipoda	Leucothoidae
*Leucothoe lilljeborgi*	102462	2	3	S	Arthropoda	Malacostraca	Amphipoda	Leucothoidae
*Leucothoe richiardii*	212784	2	3	S	Arthropoda	Malacostraca	Amphipoda	Leucothoidae
*Leucothoe spinicarpa*	102470	2	3	S	Arthropoda	Malacostraca	Amphipoda	Leucothoidae
Liljeborgia	101582	2	3	S	Arthropoda	Malacostraca	Amphipoda	Liljeborgiidae
*Liljeborgia kinahani*	102483	2	3	S	Arthropoda	Malacostraca	Amphipoda	Liljeborgiidae
*Liljeborgia pallida*	102485	2	3	S	Arthropoda	Malacostraca	Amphipoda	Liljeborgiidae
*Liljeborgia psaltrica*	102486	2	3	S	Arthropoda	Malacostraca	Amphipoda	Liljeborgiidae
*Listriella mollis*	102488	2	3	S	Arthropoda	Malacostraca	Amphipoda	Liljeborgiidae
*Hippomedon bidentatus*	102569	2	3	S	Arthropoda	Malacostraca	Amphipoda	Lysianassidae
*Hippomedon denticulatus*	102570	2	3	S	Arthropoda	Malacostraca	Amphipoda	Lysianassidae
*Hippomedon massiliensis*	102576	2	3	S	Arthropoda	Malacostraca	Amphipoda	Lysianassidae
*Lepidepecreum longicornis*	102599	2	3	S	Arthropoda	Malacostraca	Amphipoda	Lysianassidae
Lysianassidae	101395	2	3	S	Arthropoda	Malacostraca	Amphipoda	Lysianassidae
*Orchomene humilis*	102665	2	3	S	Arthropoda	Malacostraca	Amphipoda	Lysianassidae
*Orchomenella nana*	102691	2	3	S	Arthropoda	Malacostraca	Amphipoda	Lysianassidae
*Tryphosella sarsi*	102771	2	3	S	Arthropoda	Malacostraca	Amphipoda	Lysianassidae
*Tryphosella simillima*	102773	2	3	S	Arthropoda	Malacostraca	Amphipoda	Lysianassidae
*Tryphosites longipes*	102779	2	3	S	Arthropoda	Malacostraca	Amphipoda	Lysianassidae
*Acidostoma nodiferum*	102496	2	3	S	Arthropoda	Malacostraca	Amphipoda	Lysianassidae s.l.
*Acidostoma obesum*	102497	2	3	S	Arthropoda	Malacostraca	Amphipoda	Lysianassidae s.l.
*Animoceradocus semiserratus*	531364	2	3	S	Arthropoda	Malacostraca	Amphipoda	Maeridae
Maera	101675	2	3	S	Arthropoda	Malacostraca	Amphipoda	Maeridae
*Maera grossimana*	102815	2	3	S	Arthropoda	Malacostraca	Amphipoda	Maeridae
*Maera loveni*	102820	2	3	S	Arthropoda	Malacostraca	Amphipoda	Maeridae
*Maera schmidti*	102826	2	3	S	Arthropoda	Malacostraca	Amphipoda	Maeridae
*Othomaera othonis*	534781	2	3	S	Arthropoda	Malacostraca	Amphipoda	Maeridae
*Megaluropus agilis*	102783	2	3	S	Arthropoda	Malacostraca	Amphipoda	Megaluropidae
*Abludomelita obtusata*	102788	2	2	S	Arthropoda	Malacostraca	Amphipoda	Melitidae
*Eriopisa elongata*	102807	2	2	S	Arthropoda	Malacostraca	Amphipoda	Melitidae
*Maerella tenuimana*	102831	2	2	S	Arthropoda	Malacostraca	Amphipoda	Melitidae
Microprotopus	101561	2	3	S	Arthropoda	Malacostraca	Amphipoda	Microprotopidae
*Microprotopus maculatus*	102380	2	3	S	Arthropoda	Malacostraca	Amphipoda	Microprotopidae
*Odius carinatus*	102861	1	3	E	Arthropoda	Malacostraca	Amphipoda	Odiidae
*Deflexilodes acutipes*	236538	2	3	S	Arthropoda	Malacostraca	Amphipoda	Oedicerotidae
*Deflexilodes gibbosus*	236539	2	3	S	Arthropoda	Malacostraca	Amphipoda	Oedicerotidae
*Monoculodes carinatus*	102882	2	3	S	Arthropoda	Malacostraca	Amphipoda	Oedicerotidae
*Perioculodes longimanus*	102915	2	3	S	Arthropoda	Malacostraca	Amphipoda	Oedicerotidae
*Pontocrates altamarinus*	102916	2	3	S	Arthropoda	Malacostraca	Amphipoda	Oedicerotidae
*Pontocrates arenarius*	102918	2	3	S	Arthropoda	Malacostraca	Amphipoda	Oedicerotidae
*Synchelidium haplocheles*	102924	2	2	S	Arthropoda	Malacostraca	Amphipoda	Oedicerotidae
*Synchelidium maculatum*	102928	2	2	S	Arthropoda	Malacostraca	Amphipoda	Oedicerotidae
*Westwoodilla caecula*	102932	2	3	B	Arthropoda	Malacostraca	Amphipoda	Oedicerotidae
*Westwoodilla rectirostris*	102937	2	3	B	Arthropoda	Malacostraca	Amphipoda	Oedicerotidae
*Gammaropsis cornuta*	148545	2	2	S	Arthropoda	Malacostraca	Amphipoda	Photidae
*Gammaropsis maculata*	102364	2	2	S	Arthropoda	Malacostraca	Amphipoda	Photidae
*Gammaropsis nitida*	102367	1	2	S	Arthropoda	Malacostraca	Amphipoda	Photidae
*Gammaropsis palmata*	102369	2	2	S	Arthropoda	Malacostraca	Amphipoda	Photidae
*Gammaropsis sophiae*	102371	2	2	S	Arthropoda	Malacostraca	Amphipoda	Photidae
*Megamphopus cornutus*	102377	2	3	S	Arthropoda	Malacostraca	Amphipoda	Photidae
*Photis longicaudata*	102383	2	1	S	Arthropoda	Malacostraca	Amphipoda	Photidae
*Photis reinhardi*	102387	2	1	S	Arthropoda	Malacostraca	Amphipoda	Photidae
Harpinia	101716	2	3	S	Arthropoda	Malacostraca	Amphipoda	Phoxocephalidae
*Harpinia agna*	102957	2	3	S	Arthropoda	Malacostraca	Amphipoda	Phoxocephalidae
*Harpinia ala*	102958	2	3	S	Arthropoda	Malacostraca	Amphipoda	Phoxocephalidae
*Harpinia antennaria*	102960	2	3	S	Arthropoda	Malacostraca	Amphipoda	Phoxocephalidae
*Harpinia crenulata*	102963	2	3	S	Arthropoda	Malacostraca	Amphipoda	Phoxocephalidae
*Harpinia dellavallei*	102966	2	3	S	Arthropoda	Malacostraca	Amphipoda	Phoxocephalidae
*Harpinia pectinata*	102972	2	3	S	Arthropoda	Malacostraca	Amphipoda	Phoxocephalidae
*Harpinia plumosa*	102973	2	3	S	Arthropoda	Malacostraca	Amphipoda	Phoxocephalidae
Metaphoxus	101720	2	3	S	Arthropoda	Malacostraca	Amphipoda	Phoxocephalidae
*Metaphoxus pectinatus*	102983	2	3	S	Arthropoda	Malacostraca	Amphipoda	Phoxocephalidae
*Parametaphoxus tulearensis*	549079	2	3	S	Arthropoda	Malacostraca	Amphipoda	Phoxocephalidae
*Phoxocephalus holbolli*	102989	2	3	S	Arthropoda	Malacostraca	Amphipoda	Phoxocephalidae
Pleustidae	101404	2	3	S	Arthropoda	Malacostraca	Amphipoda	Pleustidae
Bathyporeia	101742	2	3	S	Arthropoda	Malacostraca	Amphipoda	Pontoporeiidae
*Bathyporeia elegans*	103058	2	3	S	Arthropoda	Malacostraca	Amphipoda	Pontoporeiidae
*Bathyporeia guilliamsoniana*	103060	2	3	S	Arthropoda	Malacostraca	Amphipoda	Pontoporeiidae
*Bathyporeia nana*	103064	2	3	S	Arthropoda	Malacostraca	Amphipoda	Pontoporeiidae
*Bathyporeia pelagica*	103066	2	3	S	Arthropoda	Malacostraca	Amphipoda	Pontoporeiidae
*Bathyporeia pilosa*	103068	2	3	S	Arthropoda	Malacostraca	Amphipoda	Pontoporeiidae
*Bathyporeia sarsi*	103073	2	3	S	Arthropoda	Malacostraca	Amphipoda	Pontoporeiidae
*Bathyporeia tenuipes*	103076	2	3	S	Arthropoda	Malacostraca	Amphipoda	Pontoporeiidae
*Scopelocheirus hopei*	102720	2	3	S	Arthropoda	Malacostraca	Amphipoda	Scopelocheiridae
*Stegocephaloides christianiensis*	103102	2	3	S	Arthropoda	Malacostraca	Amphipoda	Stegocephalidae
Metopa	101764	2	3	S	Arthropoda	Malacostraca	Amphipoda	Stenothoidae
*Metopa alderi*	103116	2	3	S	Arthropoda	Malacostraca	Amphipoda	Stenothoidae
*Metopa latimana*	103125	2	3	S	Arthropoda	Malacostraca	Amphipoda	Stenothoidae
*Stenothoe marina*	103166	2	3	S	Arthropoda	Malacostraca	Amphipoda	Stenothoidae
*Austrosyrrhoe fimbriatus*	103179	2	3	S	Arthropoda	Malacostraca	Amphipoda	Synopiidae
*Unciola crenatipalma*	102057	2	3	B	Arthropoda	Malacostraca	Amphipoda	Unciolidae
*Unciola planipes*	102061	2	3	B	Arthropoda	Malacostraca	Amphipoda	Unciolidae
*Tmetonyx cicada*	102736	2	1	B	Arthropoda	Malacostraca	Amphipoda	Uristidae
Urothoe	101789	2	3	S	Arthropoda	Malacostraca	Amphipoda	Urothoidae
*Urothoe brevicornis*	103226	2	3	S	Arthropoda	Malacostraca	Amphipoda	Urothoidae
*Urothoe elegans*	103228	2	3	S	Arthropoda	Malacostraca	Amphipoda	Urothoidae
*Urothoe marina*	103233	2	3	S	Arthropoda	Malacostraca	Amphipoda	Urothoidae
*Urothoe poseidonis*	103235	2	3	S	Arthropoda	Malacostraca	Amphipoda	Urothoidae
*Bodotria scorpioides*	110445	2	3	S	Arthropoda	Malacostraca	Cumacea	Bodotriidae
Iphinoe	110391	2	3	S	Arthropoda	Malacostraca	Cumacea	Bodotriidae
*Iphinoe serrata*	110460	2	3	S	Arthropoda	Malacostraca	Cumacea	Bodotriidae
*Iphinoe tenella*	110461	2	3	S	Arthropoda	Malacostraca	Cumacea	Bodotriidae
*Iphinoe trispinosa*	110462	2	3	S	Arthropoda	Malacostraca	Cumacea	Bodotriidae
Diastylidae	110380	2	3	S	Arthropoda	Malacostraca	Cumacea	Diastylidae
Diastylis	110398	2	3	S	Arthropoda	Malacostraca	Cumacea	Diastylidae
*Diastylis bradyi*	110472	2	3	S	Arthropoda	Malacostraca	Cumacea	Diastylidae
*Diastylis cornuta*	110474	2	3	S	Arthropoda	Malacostraca	Cumacea	Diastylidae
*Diastylis laevis*	110481	2	3	S	Arthropoda	Malacostraca	Cumacea	Diastylidae
*Diastylis lucifera*	110483	2	3	S	Arthropoda	Malacostraca	Cumacea	Diastylidae
*Diastylis neapolitana*	110484	2	3	S	Arthropoda	Malacostraca	Cumacea	Diastylidae
*Diastylis rathkei*	110487	2	3	S	Arthropoda	Malacostraca	Cumacea	Diastylidae
*Diastylis rugosa*	110488	2	3	S	Arthropoda	Malacostraca	Cumacea	Diastylidae
*Diastyloides biplicatus*	110494	2	2	S	Arthropoda	Malacostraca	Cumacea	Diastylidae
*Hemilamprops roseus*	110514	2	3	S	Arthropoda	Malacostraca	Cumacea	Lampropidae
*Lamprops fasciatus*	110516	2	3	S	Arthropoda	Malacostraca	Cumacea	Lampropidae
*Eudorella emarginata*	110524	2	3	S	Arthropoda	Malacostraca	Cumacea	Leuconidae
*Eudorella truncatula*	110535	2	3	S	Arthropoda	Malacostraca	Cumacea	Leuconidae
*Eudorellopsis deformis*	110536	2	3	S	Arthropoda	Malacostraca	Cumacea	Leuconidae
*Leucon (Leucon) nasica*	110618	2	3	S	Arthropoda	Malacostraca	Cumacea	Leuconidae
*Cumella (Cumella) pygmaea*	110567	2	3	S	Arthropoda	Malacostraca	Cumacea	Nannastacidae
*Nannastacus unguiculatus*	110574	2	3	S	Arthropoda	Malacostraca	Cumacea	Nannastacidae
*Monopseudocuma gilsoni*	422916	2	3	S	Arthropoda	Malacostraca	Cumacea	Pseudocumatidae
Pseudocuma	110427	2	3	S	Arthropoda	Malacostraca	Cumacea	Pseudocumatidae
*Pseudocuma (Pseudocuma) longicorne*	110627	2	3	S	Arthropoda	Malacostraca	Cumacea	Pseudocumatidae
*Pseudocuma (Pseudocuma) simile*	110628	2	3	S	Arthropoda	Malacostraca	Cumacea	Pseudocumatidae
*Alpheus glaber*	107477	4	4	B	Arthropoda	Malacostraca	Decapoda	Alpheidae
*Athanas nitescens*	107486	2	2	S	Arthropoda	Malacostraca	Decapoda	Alpheidae
*Aristaeomorpha foliacea*	158326	1	4	E	Arthropoda	Malacostraca	Decapoda	Aristeidae
*Atelecyclus rotundatus*	107273	5	4	R	Arthropoda	Malacostraca	Decapoda	Atelecyclidae
*Calocaris macandreae*	107726	4	4	B	Arthropoda	Malacostraca	Decapoda	Axiidae
Callianassa	107072	4	4	B	Arthropoda	Malacostraca	Decapoda	Callianassidae
*Callianassa subterranea*	107729	4	4	B	Arthropoda	Malacostraca	Decapoda	Callianassidae
Callianassidae	106800	4	4	B	Arthropoda	Malacostraca	Decapoda	Callianassidae
*Pestarella tyrrhena*	238027	4	4	B	Arthropoda	Malacostraca	Decapoda	Callianassidae
*Corystes cassivelaunus*	107277	5	4	R	Arthropoda	Malacostraca	Decapoda	Corystidae
Crangon	107007	2	4	S	Arthropoda	Malacostraca	Decapoda	Crangonidae
*Crangon allmanni*	107551	2	4	S	Arthropoda	Malacostraca	Decapoda	Crangonidae
*Crangon crangon*	107552	2	4	S	Arthropoda	Malacostraca	Decapoda	Crangonidae
Crangonidae	106782	2	4	S	Arthropoda	Malacostraca	Decapoda	Crangonidae
*Philocheras bispinosus bispinosus*	108207	2	4	S	Arthropoda	Malacostraca	Decapoda	Crangonidae
*Philocheras trispinosus*	107562	2	4	S	Arthropoda	Malacostraca	Decapoda	Crangonidae
*Diogenes pugilator*	107199	1	4	E	Arthropoda	Malacostraca	Decapoda	Diogenidae
Galathea	106834	4	3	B	Arthropoda	Malacostraca	Decapoda	Galatheidae
*Galathea dispersa*	107148	4	3	B	Arthropoda	Malacostraca	Decapoda	Galatheidae
*Galathea intermedia*	107150	4	3	B	Arthropoda	Malacostraca	Decapoda	Galatheidae
*Eualus cranchii*	156083	2	4	S	Arthropoda	Malacostraca	Decapoda	Hippolytidae
Inachus	106905	1	3	E	Arthropoda	Malacostraca	Decapoda	Inachidae
*Inachus dorsettensis*	107327	1	3	E	Arthropoda	Malacostraca	Decapoda	Inachidae
Macropodia	205077	1	3	E	Arthropoda	Malacostraca	Decapoda	Inachidae
*Macropodia deflexa*	107338	1	3	E	Arthropoda	Malacostraca	Decapoda	Inachidae
*Macropodia rostrata*	107345	1	3	E	Arthropoda	Malacostraca	Decapoda	Inachidae
*Jaxea nocturna*	107737	4	4	B	Arthropoda	Malacostraca	Decapoda	Laomediidae
Ebalia	106889	1	3	E	Arthropoda	Malacostraca	Decapoda	Leucosiidae
*Ebalia cranchii*	107294	1	3	E	Arthropoda	Malacostraca	Decapoda	Leucosiidae
*Ebalia granulosa*	107298	1	3	E	Arthropoda	Malacostraca	Decapoda	Leucosiidae
*Ebalia tuberosa*	107301	1	3	E	Arthropoda	Malacostraca	Decapoda	Leucosiidae
*Nephrops norvegicus*	107254	4	4	B	Arthropoda	Malacostraca	Decapoda	Nephropidae
Anapagurus	106849	1	4	E	Arthropoda	Malacostraca	Decapoda	Paguridae
*Anapagurus bicorniger*	107213	1	4	E	Arthropoda	Malacostraca	Decapoda	Paguridae
*Anapagurus laevis*	107218	1	4	E	Arthropoda	Malacostraca	Decapoda	Paguridae
Paguridae	106738	1	4	E	Arthropoda	Malacostraca	Decapoda	Paguridae
Pagurus	106854	1	4	E	Arthropoda	Malacostraca	Decapoda	Paguridae
*Pagurus bernhardus*	107232	1	4	E	Arthropoda	Malacostraca	Decapoda	Paguridae
*Pagurus cuanensis*	107235	1	4	E	Arthropoda	Malacostraca	Decapoda	Paguridae
*Palaemon elegans*	107614	1	4	E	Arthropoda	Malacostraca	Decapoda	Palaemonidae
*Pandalus montagui*	107651	1	4	E	Arthropoda	Malacostraca	Decapoda	Pandalidae
*Pilumnus hirtellus*	107418	2	3	S	Arthropoda	Malacostraca	Decapoda	Pilumnidae
Liocarcinus	106925	5	4	R	Arthropoda	Malacostraca	Decapoda	Polybiidae
*Liocarcinus depurator*	107387	5	4	R	Arthropoda	Malacostraca	Decapoda	Polybiidae
*Liocarcinus holsatus*	107388	5	4	R	Arthropoda	Malacostraca	Decapoda	Polybiidae
*Liocarcinus marmoreus*	107390	5	4	R	Arthropoda	Malacostraca	Decapoda	Polybiidae
*Liocarcinus navigator*	107392	5	4	R	Arthropoda	Malacostraca	Decapoda	Polybiidae
*Liocarcinus pusillus*	107393	5	4	R	Arthropoda	Malacostraca	Decapoda	Polybiidae
*Pisidia longicornis*	107188	1	3	E	Arthropoda	Malacostraca	Decapoda	Porcellanidae
*Carcinus maenas*	107381	5	4	R	Arthropoda	Malacostraca	Decapoda	Portunidae
Processa	107054	1	4	E	Arthropoda	Malacostraca	Decapoda	Processidae
*Processa acutirostris*	107681	1	4	E	Arthropoda	Malacostraca	Decapoda	Processidae
*Processa canaliculata*	107682	1	4	E	Arthropoda	Malacostraca	Decapoda	Processidae
*Processa elegantula*	107684	1	4	E	Arthropoda	Malacostraca	Decapoda	Processidae
*Processa modica modica*	108343	1	4	E	Arthropoda	Malacostraca	Decapoda	Processidae
*Processa nouveli holthuisi*	108344	1	4	E	Arthropoda	Malacostraca	Decapoda	Processidae
*Solenocera membranacea*	107120	1	4	E	Arthropoda	Malacostraca	Decapoda	Solenoceridae
*Thia scutellata*	107281	2	4	S	Arthropoda	Malacostraca	Decapoda	Thiidae
*Upogebia deltaura*	107739	3	4	DC	Arthropoda	Malacostraca	Decapoda	Upogebiidae
*Upogebia pusilla*	107741	3	4	DC	Arthropoda	Malacostraca	Decapoda	Upogebiidae
*Upogebia stellata*	107742	3	4	DC	Arthropoda	Malacostraca	Decapoda	Upogebiidae
Upogebiidae	106803	3	4	DC	Arthropoda	Malacostraca	Decapoda	Upogebiidae
*Anthura gracilis*	118467	2	2	S	Arthropoda	Malacostraca	Isopoda	Anthuridae
*Astacilla longicornis*	119024	4	3	B	Arthropoda	Malacostraca	Isopoda	Arcturidae
*Cirolana cranchi*	118839	2	3	S	Arthropoda	Malacostraca	Isopoda	Cirolanidae
*Conilera cylindracea*	118842	1	2	E	Arthropoda	Malacostraca	Isopoda	Cirolanidae
*Eurydice pulchra*	118852	2	3	S	Arthropoda	Malacostraca	Isopoda	Cirolanidae
*Eurydice spinigera*	148637	2	3	S	Arthropoda	Malacostraca	Isopoda	Cirolanidae
*Natatolana borealis*	118859	2	3	S	Arthropoda	Malacostraca	Isopoda	Cirolanidae
*Gnathia maxillaris*	118994	2	3	S	Arthropoda	Malacostraca	Isopoda	Gnathiidae
*Gnathia oxyuraea*	118995	2	3	S	Arthropoda	Malacostraca	Isopoda	Gnathiidae
Gnathiidae	118278	2	3	S	Arthropoda	Malacostraca	Isopoda	Gnathiidae
*Paragnathia formica*	119001	2	3	S	Arthropoda	Malacostraca	Isopoda	Gnathiidae
Idotea	118454	2	3	S	Arthropoda	Malacostraca	Isopoda	Idoteidae
*Janira maculosa*	118732	2	3	S	Arthropoda	Malacostraca	Isopoda	Janiridae
*Sphaeroma serratum*	118973	2	3	S	Arthropoda	Malacostraca	Isopoda	Sphaeromatidae
*Gastrosaccus spinifer*	120020	2	3	S	Arthropoda	Malacostraca	Mysida	Mysidae
*Haplostylus normani*	148698	1	1	E	Arthropoda	Malacostraca	Mysida	Mysidae
*Nebalia bipes*	147032	1	3	E	Arthropoda	Malacostraca	Nebaliacea	Nebaliidae
*Apseudes spinosus*	136284	2	2	S	Arthropoda	Malacostraca	Tanaidacea	Apseudidae
*Apseudes talpa*	136285	2	2	S	Arthropoda	Malacostraca	Tanaidacea	Apseudidae
*Apseudopsis latreillii*	247077	2	2	S	Arthropoda	Malacostraca	Tanaidacea	Apseudidae
*Akanthophoreus gracilis*	136340	2	2	S	Arthropoda	Malacostraca	Tanaidacea	Leptognathiidae
*Pseudoparatanais batei*	136457	2	2	S	Arthropoda	Malacostraca	Tanaidacea	Paratanaoidea incertae sedis
*Tanaopsis graciloides*	136458	2	2	S	Arthropoda	Malacostraca	Tanaidacea	Paratanaoidea incertae sedis
*Pseudosphyrapus anomalus*	136319	2	2	S	Arthropoda	Malacostraca	Tanaidacea	Sphyrapidae
*Tanaissus lilljeborgi*	136486	2	2	S	Arthropoda	Malacostraca	Tanaidacea	Tanaissuidae
Stomatopoda	14355	4	4	B	Arthropoda	Malacostraca		
*Longipedia coronata*	116368	2	3	S	Arthropoda	Maxillopoda	Harpacticoida	Longipediidae
*Cylindroleberis mariae*	238708	2	3	S	Arthropoda	Ostracoda	Myodocopida	Cylindroleberididae
*Philomedes (Philomedes) lilljeborgi*	128460	2	4	S	Arthropoda	Ostracoda	Myodocopida	Philomedidae
*Philomedes brenda*	127718	2	3	S	Arthropoda	Ostracoda	Myodocopida	Philomedidae
Myodocopida	2104	2	3	S	Arthropoda	Ostracoda	Myodocopida	
*Achelia echinata*	134599	2	2	S	Arthropoda	Pycnogonida	Pantopoda	Ammotheidae
*Callipallene brevirostris*	134643	1	1	E	Arthropoda	Pycnogonida	Pantopoda	Callipallenidae
*Nymphon brevirostre*	150520	1	1	S	Arthropoda	Pycnogonida	Pantopoda	Nymphonidae
Anoplodactylus	134592	1	4	E	Arthropoda	Pycnogonida	Pantopoda	Phoxichilidiidae
*Anoplodactylus petiolatus*	134723	1	4	E	Arthropoda	Pycnogonida	Pantopoda	Phoxichilidiidae
*Anoplodactylus virescens*	134730	1	4	E	Arthropoda	Pycnogonida	Pantopoda	Phoxichilidiidae
Pycnogonida	1302	1	4	E	Arthropoda	Pycnogonida		
*Priapulus caudatus*	101160	4	2	B	Cephalorhyncha	Priapulida		Priapulidae
*Ascidiella aspersa*	103718	1	1	E	Chordata	Ascidiacea	Phlebobranchia	Ascidiidae
*Eugyra arenosa*	103764	2	3	S	Chordata	Ascidiacea	Stolidobranchia	Molgulidae
Styelidae	103450	1	1	E	Chordata	Ascidiacea	Stolidobranchia	Styelidae
*Branchiostoma lanceolatum*	104906	4	3	B	Chordata	Leptocardii		Branchiostomidae
*Actinia equina*	100803	1	1	E	Cnidaria	Anthozoa	Actiniaria	Actiniidae
*Urticina felina*	100834	1	1	E	Cnidaria	Anthozoa	Actiniaria	Actiniidae
Edwardsia	100730	2	2	S	Cnidaria	Anthozoa	Actiniaria	Edwardsiidae
*Edwardsia claparedii*	100880	2	2	S	Cnidaria	Anthozoa	Actiniaria	Edwardsiidae
Edwardsiidae	100665	2	2	S	Cnidaria	Anthozoa	Actiniaria	Edwardsiidae
*Scolanthus callimorphus*	100910	2	2	S	Cnidaria	Anthozoa	Actiniaria	Edwardsiidae
*Halcampa chrysanthellum*	100916	2	2	S	Cnidaria	Anthozoa	Actiniaria	Halcampidae
*Calliactis parasitica*	100946	2	2	S	Cnidaria	Anthozoa	Actiniaria	Hormathiidae
*Sagartia troglodytes*	100994	1	1	E	Cnidaria	Anthozoa	Actiniaria	Sagartiidae
Actiniaria	1360	2	2	S	Cnidaria	Anthozoa	Actiniaria	
Cerianthus	100782	2	1	S	Cnidaria	Anthozoa	Ceriantharia	Cerianthidae
*Cerianthus lloydii*	283798	2	1	S	Cnidaria	Anthozoa	Ceriantharia	Cerianthidae
*Pteroeides griseum*	181504	2	2	S	Cnidaria	Anthozoa	Pennatulacea	Pennatulidae
*Virgularia mirabilis*	128539	2	2	S	Cnidaria	Anthozoa	Pennatulacea	Virgulariidae
*Asterias rubens*	123776	1	3	E	Echinodermata	Asteroidea	Forcipulatida	Asteriidae
*Astropecten irregularis*	123867	2	3	S	Echinodermata	Asteroidea	Paxillosida	Astropectinidae
*Psammechinus miliaris*	124319	1	3	E	Echinodermata	Echinoidea	Camarodonta	Parechinidae
*Echinocyamus pusillus*	124273	2	3	S	Echinodermata	Echinoidea	Clypeasteroida	Echinocyamidae
Echinidea	510534	4	3	B	Echinodermata	Echinoidea	Echinoida	
*Brissopsis lyrifera*	124373	4	3	B	Echinodermata	Echinoidea	Spatangoida	Brissidae
Echinocardium	123426	4	3	B	Echinodermata	Echinoidea	Spatangoida	Loveniidae
*Echinocardium cordatum*	124392	4	3	B	Echinodermata	Echinoidea	Spatangoida	Loveniidae
*Echinocardium flavescens*	124394	4	3	B	Echinodermata	Echinoidea	Spatangoida	Loveniidae
*Spatangus purpureus*	124418	4	3	B	Echinodermata	Echinoidea	Spatangoida	Spatangidae
Spatangoida	123106	4	3	B	Echinodermata	Echinoidea	Spatangoida	
*Labidoplax buskii*	124455	2	3	S	Echinodermata	Holothuroidea	Apodida	Synaptidae
Leptosynapta	123449	2	3	S	Echinodermata	Holothuroidea	Apodida	Synaptidae
*Leptosynapta bergensis*	124462	2	3	S	Echinodermata	Holothuroidea	Apodida	Synaptidae
*Leptosynapta inhaerens*	124465	2	3	S	Echinodermata	Holothuroidea	Apodida	Synaptidae
*Oestergrenia digitata*	152547	2	3	S	Echinodermata	Holothuroidea	Apodida	Synaptidae
Synaptidae	123182	2	3	S	Echinodermata	Holothuroidea	Apodida	Synaptidae
Leptopentacta	123481	2	3	S	Echinodermata	Holothuroidea	Dendrochirotida	Cucumariidae
*Leptopentacta elongata*	124635	2	3	S	Echinodermata	Holothuroidea	Dendrochirotida	Cucumariidae
*Leptopentacta elongata*	124635	2	3	S	Echinodermata	Holothuroidea	Dendrochirotida	Cucumariidae
*Leptopentacta tergestina*	124636	2	3	S	Echinodermata	Holothuroidea	Dendrochirotida	Cucumariidae
*Thyone fusus*	124670	2	3	S	Echinodermata	Holothuroidea	Dendrochirotida	Cucumariidae
*Amphilepis norvegica*	125057	4	3	B	Echinodermata	Ophiuroidea	Ophiurida	Amphilepididae
*Acrocnida brachiata*	236130	4	3	B	Echinodermata	Ophiuroidea	Ophiurida	Amphiuridae
*Amphipholis squamata*	125064	4	3	B	Echinodermata	Ophiuroidea	Ophiurida	Amphiuridae
Amphiura	123613	4	3	B	Echinodermata	Ophiuroidea	Ophiurida	Amphiuridae
*Amphiura (Ophiopeltis) securigera*	125195	4	3	B	Echinodermata	Ophiuroidea	Ophiurida	Amphiuridae
*Amphiura chiajei*	125073	4	3	B	Echinodermata	Ophiuroidea	Ophiurida	Amphiuridae
*Amphiura filiformis*	125080	4	3	B	Echinodermata	Ophiuroidea	Ophiurida	Amphiuridae
*Ophiocomina nigra*	125027	2	2	S	Echinodermata	Ophiuroidea	Ophiurida	Ophiocomidae
*Ophiothrix fragilis*	125131	2	2	S	Echinodermata	Ophiuroidea	Ophiurida	Ophiotrichidae
*Ophiocten affinis*	124850	2	2	S	Echinodermata	Ophiuroidea	Ophiurida	Ophiuridae
Ophiura	123574	2	2	S	Echinodermata	Ophiuroidea	Ophiurida	Ophiuridae
*Ophiura albida*	124913	2	2	S	Echinodermata	Ophiuroidea	Ophiurida	Ophiuridae
*Ophiura ophiura*	124929	2	2	S	Echinodermata	Ophiuroidea	Ophiurida	Ophiuridae
Ophiuridae	123200	2	2	S	Echinodermata	Ophiuroidea	Ophiurida	Ophiuridae
*Maxmuelleria gigas*	110367	5	4	R	Echiura	Echiuroidea	Bonelliida	Bonelliidae
*Echiurus echiurus*	110377	3	2	DC	Echiura	Echiuroidea	Echiurida	Echiuridae
*Thalassema thalassemum*	110375	3	2	DC	Echiura	Echiuroidea	Echiurida	Echiuridae
Echiurida	110345	3	2	DC	Echiura	Echiuroidea	Echiurida	
Echiura	1269	3	2	DC	Echiura			
*Glandiceps talaboti*	137612	5	4	R	HemiChordata	Enteropneusta	Enteropneusta	Spengelidae
Enteroptneusta	178738	5	4	R	HemiChordata	Enteropneusta		
*Ensis magnus*	160539	2	2	S	Mollusca	Bivalvia	[unassigned] Euheterodonta	Pharidae
*Hiatella arctica*	140103	1	2	E	Mollusca	Bivalvia	[unassigned] Euheterodonta	Hiatellidae
*Saxicavella jeffreysi*	140108	1	2	E	Mollusca	Bivalvia	[unassigned] Euheterodonta	Hiatellidae
Ensis	138333	2	2	S	Mollusca	Bivalvia	[unassigned] Euheterodonta	Pharidae
*Ensis directus*	140732	2	2	S	Mollusca	Bivalvia	[unassigned] Euheterodonta	Pharidae
*Ensis ensis*	140733	2	2	S	Mollusca	Bivalvia	[unassigned] Euheterodonta	Pharidae
*Pharus legumen*	140736	2	2	S	Mollusca	Bivalvia	[unassigned] Euheterodonta	Pharidae
*Phaxas adriaticus*	156343	2	2	S	Mollusca	Bivalvia	[unassigned] Euheterodonta	Pharidae
*Phaxas pellucidus*	140737	2	2	S	Mollusca	Bivalvia	[unassigned] Euheterodonta	Pharidae
*Cuspidaria obesa*	139450	3	2	UC/DC	Mollusca	Bivalvia	Anomalodesmata	Cuspidariidae
*Lyonsia norwegica*	140291	2	2	S	Mollusca	Bivalvia	Anomalodesmata	Lyonsiidae
*Cochlodesma praetenue*	181373	3	2	DC	Mollusca	Bivalvia	Anomalodesmata	Periplomatidae
Thracia	138549	3	2	DC	Mollusca	Bivalvia	Anomalodesmata	Thraciidae
*Thracia convexa*	141644	3	2	DC	Mollusca	Bivalvia	Anomalodesmata	Thraciidae
*Thracia phaseolina*	152378	3	2	DC	Mollusca	Bivalvia	Anomalodesmata	Thraciidae
*Thracia villosiuscula*	141651	3	2	DC	Mollusca	Bivalvia	Anomalodesmata	Thraciidae
*Glycymeris glycymeris*	140025	2	2	S	Mollusca	Bivalvia	Arcoida	Glycymerididae
*Limopsis cristata*	140253	1	1	E	Mollusca	Bivalvia	Arcoida	Limopsidae
*Astarte borealis*	138818	2	2	S	Mollusca	Bivalvia	Carditoida	Astartidae
*Astarte montagui*	138823	2	2	S	Mollusca	Bivalvia	Carditoida	Astartidae
*Goodallia triangularis*	138831	2	2	S	Mollusca	Bivalvia	Carditoida	Astartidae
Arcturellina	492197	2	2	S	Mollusca	Bivalvia	Carditoida	Carditidae
*Limatula gwyni*	140237	1	2	E	Mollusca	Bivalvia	Limoida	Limidae
*Limatula subauriculata*	140242	1	2	E	Mollusca	Bivalvia	Limoida	Limidae
*Lucinella divaricata*	140282	2	2	S	Mollusca	Bivalvia	Lucinoida	Lucinidae
*Lucinoma borealis*	140283	2	2	S	Mollusca	Bivalvia	Lucinoida	Lucinidae
*Myrtea spinifera*	140287	2	2	S	Mollusca	Bivalvia	Lucinoida	Lucinidae
*Axinulus croulinensis*	234161	3	2	DC	Mollusca	Bivalvia	Lucinoida	Thyasiridae
*Axinulus eumyarius*	234162	3	2	UC/DC	Mollusca	Bivalvia	Lucinoida	Thyasiridae
*Mendicula ferruginosa*	152905	2	2	S	Mollusca	Bivalvia	Lucinoida	Thyasiridae
*Mendicula pygmaea*	152424	2	2	S	Mollusca	Bivalvia	Lucinoida	Thyasiridae
Thyasira	138552	3	2	DC	Mollusca	Bivalvia	Lucinoida	Thyasiridae
*Thyasira biplicata*	141655	3	2	DC	Mollusca	Bivalvia	Lucinoida	Thyasiridae
*Thyasira equalis*	141659	3	2	DC	Mollusca	Bivalvia	Lucinoida	Thyasiridae
*Thyasira flexuosa*	141662	3	2	DC	Mollusca	Bivalvia	Lucinoida	Thyasiridae
*Thyasira obsoleta*	141668	3	2	DC	Mollusca	Bivalvia	Lucinoida	Thyasiridae
*Thyasira sarsi*	141672	3	2	DC	Mollusca	Bivalvia	Lucinoida	Thyasiridae
*Thyasira succisa*	141676	3	2	DC	Mollusca	Bivalvia	Lucinoida	Thyasiridae
*Corbula gibba*	139410	2	2	S	Mollusca	Bivalvia	Myoida	Corbulidae
Mya	138211	2	2	S	Mollusca	Bivalvia	Myoida	Myidae
*Mya arenaria*	140430	2	2	S	Mollusca	Bivalvia	Myoida	Myidae
*Mya truncata*	140431	2	2	S	Mollusca	Bivalvia	Myoida	Myidae
*Sphenia binghami*	140432	2	2	S	Mollusca	Bivalvia	Myoida	Myidae
*Crenella decussata*	140440	2	3	S	Mollusca	Bivalvia	Mytiloida	Mytilidae
*Modiolula phaseolina*	140461	2	2	S	Mollusca	Bivalvia	Mytiloida	Mytilidae
*Modiolus modiolus*	140467	2	2	S	Mollusca	Bivalvia	Mytiloida	Mytilidae
Mytilus	138228	1	1	E	Mollusca	Bivalvia	Mytiloida	Mytilidae
*Mytilus edulis*	140480	1	1	E	Mollusca	Bivalvia	Mytiloida	Mytilidae
*Nuculana minuta*	140577	2	3	S	Mollusca	Bivalvia	Nuculanoida	Nuculanidae
*Yoldiella lucida*	142002	2	3	S	Mollusca	Bivalvia	Nuculanoida	Yoldiidae
*Ennucula tenuis*	140584	2	3	S	Mollusca	Bivalvia	Nuculida	Nuculidae
Nucula	138262	2	3	S	Mollusca	Bivalvia	Nuculida	Nuculidae
*Nucula hanleyi*	140588	2	3	S	Mollusca	Bivalvia	Nuculida	Nuculidae
*Nucula nitidosa*	140589	2	3	S	Mollusca	Bivalvia	Nuculida	Nuculidae
*Nucula nucleus*	140590	2	3	S	Mollusca	Bivalvia	Nuculida	Nuculidae
*Nucula sulcata*	140592	2	3	S	Mollusca	Bivalvia	Nuculida	Nuculidae
*Nucula turgida*	152274	2	3	S	Mollusca	Bivalvia	Nuculida	Nuculidae
Nuculidae	204	2	3	S	Mollusca	Bivalvia	Nuculida	Nuculidae
Anomiidae	214	1	1	E	Mollusca	Bivalvia	Pectinoida	Anomiidae
*Monia patelliformis*	153027	2	2	S	Mollusca	Bivalvia	Pectinoida	Anomiidae
*Aequipecten opercularis*	140687	2	3	S	Mollusca	Bivalvia	Pectinoida	Pectinidae
*Palliolum striatum*	140709	2	3	S	Mollusca	Bivalvia	Pectinoida	Pectinidae
*Palliolum tigerinum*	140710	2	3	S	Mollusca	Bivalvia	Pectinoida	Pectinidae
Pectinidae	213	2	3	S	Mollusca	Bivalvia	Pectinoida	Pectinidae
*Arctica islandica*	138802	2	2	S	Mollusca	Bivalvia	Veneroida	Arcticidae
Acanthocardia	137732	2	2	S	Mollusca	Bivalvia	Veneroida	Cardiidae
*Acanthocardia aculeata*	138990	2	2	S	Mollusca	Bivalvia	Veneroida	Cardiidae
*Acanthocardia echinata*	138992	2	2	S	Mollusca	Bivalvia	Veneroida	Cardiidae
*Acanthocardia paucicostata*	138993	2	2	S	Mollusca	Bivalvia	Veneroida	Cardiidae
Cardiidae	229	2	2	S	Mollusca	Bivalvia	Veneroida	Cardiidae
*Cerastoderma edule*	138998	2	2	S	Mollusca	Bivalvia	Veneroida	Cardiidae
*Laevicardium crassum*	139004	2	2	S	Mollusca	Bivalvia	Veneroida	Cardiidae
*Parvicardium exiguum*	139008	2	2	S	Mollusca	Bivalvia	Veneroida	Cardiidae
*Parvicardium minimum*	139010	2	2	S	Mollusca	Bivalvia	Veneroida	Cardiidae
*Parvicardium scabrum*	139012	2	2	S	Mollusca	Bivalvia	Veneroida	Cardiidae
*Donax vittatus*	139604	2	2	S	Mollusca	Bivalvia	Veneroida	Donacidae
*Kelliella miliaris*	152396	4	2	B	Mollusca	Bivalvia	Veneroida	Kelliellidae
*Vesicomya abyssicola*	464333	4	2	B	Mollusca	Bivalvia	Veneroida	Kelliellidae
*Hemilepton nitidum*	246148	2	2	S	Mollusca	Bivalvia	Veneroida	Lasaeidae
*Lepton squamosum*	140218	2	2	S	Mollusca	Bivalvia	Veneroida	Lasaeidae
*Lutraria lutraria*	140295	2	2	S	Mollusca	Bivalvia	Veneroida	Mactridae
Mactra	138158	2	2	S	Mollusca	Bivalvia	Veneroida	Mactridae
*Mactra stultorum*	140299	2	2	S	Mollusca	Bivalvia	Veneroida	Mactridae
Spisula	138159	2	2	S	Mollusca	Bivalvia	Veneroida	Mactridae
*Spisula elliptica*	140300	2	2	S	Mollusca	Bivalvia	Veneroida	Mactridae
*Spisula solida*	140301	2	2	S	Mollusca	Bivalvia	Veneroida	Mactridae
*Spisula subtruncata*	140302	2	2	S	Mollusca	Bivalvia	Veneroida	Mactridae
*Devonia perrieri*	140365	2	2	S	Mollusca	Bivalvia	Veneroida	Montacutidae
*Epilepton clarkiae*	140366	2	2	S	Mollusca	Bivalvia	Veneroida	Montacutidae
*Kurtiella bidentata*	345281	2	2	S	Mollusca	Bivalvia	Veneroida	Montacutidae
*Montacuta phascolionis*	140374	2	2	S	Mollusca	Bivalvia	Veneroida	Montacutidae
*Montacuta substriata*	140377	2	2	S	Mollusca	Bivalvia	Veneroida	Montacutidae
*Tellimya ferruginosa*	146952	2	2	S	Mollusca	Bivalvia	Veneroida	Montacutidae
*Tellimya tenella*	152397	2	2	S	Mollusca	Bivalvia	Veneroida	Montacutidae
Gari	138388	2	2	S	Mollusca	Bivalvia	Veneroida	Psammobiidae
*Gari costulata*	140868	2	2	S	Mollusca	Bivalvia	Veneroida	Psammobiidae
*Gari depressa*	140869	2	2	S	Mollusca	Bivalvia	Veneroida	Psammobiidae
*Gari fervensis*	140870	2	2	S	Mollusca	Bivalvia	Veneroida	Psammobiidae
*Gari tellinella*	140873	2	2	S	Mollusca	Bivalvia	Veneroida	Psammobiidae
Abra	138474	2	2	S	Mollusca	Bivalvia	Veneroida	Semelidae
*Abra alba*	141433	2	2	S	Mollusca	Bivalvia	Veneroida	Semelidae
*Abra nitida*	141435	2	2	S	Mollusca	Bivalvia	Veneroida	Semelidae
*Abra prismatica*	141436	2	2	S	Mollusca	Bivalvia	Veneroida	Semelidae
*Azorinus chamasolen*	141541	2	2	S	Mollusca	Bivalvia	Veneroida	Solecurtidae
*Solecurtus scopula*	141543	2	2	S	Mollusca	Bivalvia	Veneroida	Solecurtidae
Angulus	146491	2	2	S	Mollusca	Bivalvia	Veneroida	Tellinidae
*Angulus fabula*	152829	2	2	S	Mollusca	Bivalvia	Veneroida	Tellinidae
*Angulus incarnatus*	594114	2	2	S	Mollusca	Bivalvia	Veneroida	Tellinidae
*Angulus pygmaeus*	152828	2	2	S	Mollusca	Bivalvia	Veneroida	Tellinidae
*Angulus tenuis*	146492	2	2	S	Mollusca	Bivalvia	Veneroida	Tellinidae
*Arcopagia crassa*	141577	2	2	S	Mollusca	Bivalvia	Veneroida	Tellinidae
*Macoma balthica*	141579	2	2	S	Mollusca	Bivalvia	Veneroida	Tellinidae
*Moerella donacina*	147021	2	2	S	Mollusca	Bivalvia	Veneroida	Tellinidae
*Peronaea planata*	605934	2	2	S	Mollusca	Bivalvia	Veneroida	Tellinidae
*Tellina distorta*	141585	2	2	S	Mollusca	Bivalvia	Veneroida	Tellinidae
*Tellina nitida*	141589	2	2	S	Mollusca	Bivalvia	Veneroida	Tellinidae
*Turtonia minuta*	141875	2	2	S	Mollusca	Bivalvia	Veneroida	Turtoniidae
*Chamelea gallina*	141907	2	2	S	Mollusca	Bivalvia	Veneroida	Veneridae
*Chamelea striatula*	141908	2	2	S	Mollusca	Bivalvia	Veneroida	Veneridae
*Clausinella fasciata*	141909	2	2	S	Mollusca	Bivalvia	Veneroida	Veneridae
Dosinia	138636	2	2	S	Mollusca	Bivalvia	Veneroida	Veneridae
*Dosinia exoleta*	141911	2	2	S	Mollusca	Bivalvia	Veneroida	Veneridae
*Dosinia lupinus*	141912	2	2	S	Mollusca	Bivalvia	Veneroida	Veneridae
*Gouldia minima*	141916	2	2	S	Mollusca	Bivalvia	Veneroida	Veneridae
*Mysia undata*	140728	2	2	S	Mollusca	Bivalvia	Veneroida	Veneridae
*Petricolaria pholadiformis*	156961	2	2	S	Mollusca	Bivalvia	Veneroida	Veneridae
*Polititapes virgineus*	507877	2	2	S	Mollusca	Bivalvia	Veneroida	Veneridae
*Timoclea ovata*	141929	2	2	S	Mollusca	Bivalvia	Veneroida	Veneridae
*Venerupis corrugata*	181364	2	2	S	Mollusca	Bivalvia	Veneroida	Veneridae
*Venerupis corrugata*	181364	2	2	S	Mollusca	Bivalvia	Veneroida	Veneridae
*Venus casina*	141934	2	2	S	Mollusca	Bivalvia	Veneroida	Veneridae
*Chaetoderma nitidulum*	139106	2	2	S	Mollusca	Caudofoveata	Chaetodermatida	Chaetodermatidae
Caudofoveata	151365	2	2	S	Mollusca	Caudofoveata		
*Odostomia scalaris*	141014	4	3	B	Mollusca	Gastropoda		Pyramidellidae
*Gibbula cineraria*	141782	1	3	E	Mollusca	Gastropoda		Trochidae
*Bittium reticulatum*	139054	2	4	S	Mollusca	Gastropoda	[unassigned] Caenogastropoda	Cerithiidae
*Epitonium turtonis*	139738	2	2	S	Mollusca	Gastropoda	[unassigned] Caenogastropoda	Epitoniidae
*Eulima bilineata*	566940	4	3	B	Mollusca	Gastropoda	[unassigned] Caenogastropoda	Eulimidae
*Eulima glabra*	139805	4	3	B	Mollusca	Gastropoda	[unassigned] Caenogastropoda	Eulimidae
Eulimidae	135	4	3	B	Mollusca	Gastropoda	[unassigned] Caenogastropoda	Eulimidae
*Vitreolina philippi*	139903	4	3	B	Mollusca	Gastropoda	[unassigned] Caenogastropoda	Eulimidae
*Turritella communis*	141872	4	3	B	Mollusca	Gastropoda	[unassigned] Caenogastropoda	Turritellidae
*Hedylopsis spiculifera*	140085	4	3	B	Mollusca	Gastropoda	Acochlidiacea	Hedylopsidae
*Akera bullata*	138734	2	3	S	Mollusca	Gastropoda	Anaspidea	Akeridae
*Cylichna alba*	139474	2	3	S	Mollusca	Gastropoda	Cephalaspidea	Cylichnidae
*Cylichna cylindracea*	139476	2	3	S	Mollusca	Gastropoda	Cephalaspidea	Cylichnidae
Philine	138339	2	3	S	Mollusca	Gastropoda	Cephalaspidea	Philinidae
*Philine aperta*	140744	2	3	S	Mollusca	Gastropoda	Cephalaspidea	Philinidae
*Philine scabra*	140761	2	3	S	Mollusca	Gastropoda	Cephalaspidea	Philinidae
Retusa	138432	2	3	S	Mollusca	Gastropoda	Cephalaspidea	Retusidae
*Retusa truncatula*	141138	2	3	S	Mollusca	Gastropoda	Cephalaspidea	Retusidae
*Roxania utriculus*	139486	2	3	S	Mollusca	Gastropoda	Cephalaspidea	Scaphandridae
*Scaphander lignarius*	139488	2	3	S	Mollusca	Gastropoda	Cephalaspidea	Scaphandridae
*Aporrhais pespelecani*	138760	2	3	S	Mollusca	Gastropoda	Littorinimorpha	Aporrhaidae
*Caecum glabrum*	138952	1	2	E	Mollusca	Gastropoda	Littorinimorpha	Caecidae
*Crepidula fornicata*	138963	1	1	E	Mollusca	Gastropoda	Littorinimorpha	Calyptraeidae
*Galeodea echinophora*	139023	1	4	E	Mollusca	Gastropoda	Littorinimorpha	Cassidae
Hydrobiidae	120	2	3	S	Mollusca	Gastropoda	Littorinimorpha	Hydrobiidae
Ceratia	138082	2	3	S	Mollusca	Gastropoda	Littorinimorpha	Iravadiidae
*Ceratia proxima*	140128	2	3	S	Mollusca	Gastropoda	Littorinimorpha	Iravadiidae
*Hyala vitrea*	140129	2	3	S	Mollusca	Gastropoda	Littorinimorpha	Iravadiidae
*Euspira pulchella*	140539	2	3	S	Mollusca	Gastropoda	Littorinimorpha	Naticidae
*Lunatia catena*	150590	2	3	S	Mollusca	Gastropoda	Littorinimorpha	Naticidae
*Lunatia montagui*	150639	2	3	S	Mollusca	Gastropoda	Littorinimorpha	Naticidae
Naticidae	145	2	3	S	Mollusca	Gastropoda	Littorinimorpha	Naticidae
Alvania	138439	2	3	S	Mollusca	Gastropoda	Littorinimorpha	Rissoidae
*Alvania cancellata*	141165	2	3	S	Mollusca	Gastropoda	Littorinimorpha	Rissoidae
*Alvania cimicoides*	141168	2	3	S	Mollusca	Gastropoda	Littorinimorpha	Rissoidae
*Alvania subsoluta*	141247	2	3	S	Mollusca	Gastropoda	Littorinimorpha	Rissoidae
*Alvania testae*	141251	2	3	S	Mollusca	Gastropoda	Littorinimorpha	Rissoidae
Rissoa	138456	2	3	S	Mollusca	Gastropoda	Littorinimorpha	Rissoidae
*Rissoa parva*	141365	2	3	S	Mollusca	Gastropoda	Littorinimorpha	Rissoidae
*Lamellaria latens*	140172	2	3	S	Mollusca	Gastropoda	Littorinimorpha	Velutinidae
*Buccinum undatum*	138878	2	4	S	Mollusca	Gastropoda	Neogastropoda	Buccinidae
*Neptunea antiqua*	138920	2	4	S	Mollusca	Gastropoda	Neogastropoda	Buccinidae
*Mangelia attenuata*	139265	2	4	S	Mollusca	Gastropoda	Neogastropoda	Conidae
*Raphitoma linearis*	139371	2	4	S	Mollusca	Gastropoda	Neogastropoda	Conidae
*Nassarius incrassatus*	140503	2	3	S	Mollusca	Gastropoda	Neogastropoda	Nassariidae
*Nassarius reticulatus*	140513	2	3	S	Mollusca	Gastropoda	Neogastropoda	Nassariidae
Onchidoris	138288	1	1	S	Mollusca	Gastropoda	Nudibranchia	Onchidorididae
Cuthona	138543	1	2	E	Mollusca	Gastropoda	Nudibranchia	Tergipedidae
*Acteon tornatilis*	138691	2	3	S	Mollusca	Gastropoda		Acteonidae
Turbonilla	138421	2	2	S	Mollusca	Gastropoda		Pyramidellidae
*Turbonilla acuta*	141052	2	2	S	Mollusca	Gastropoda		Pyramidellidae
*Turbonilla crenata*	141057	2	2	S	Mollusca	Gastropoda		Pyramidellidae
Rissoella	144173	2	3	S	Mollusca	Gastropoda		Rissoellidae
*Rissoella diaphana*	141147	2	3	S	Mollusca	Gastropoda		Rissoellidae
*Rissoella opalina*	141149	2	3	S	Mollusca	Gastropoda		Rissoellidae
*Lepidochitona (Lepidochitona) cinerea*	140143	1	2	E	Mollusca	Polyplacophora	Chitonida	Tonicellidae
*Leptochiton asellus*	140199	1	2	E	Mollusca	Polyplacophora	Lepidopleurida	Leptochitonidae
*Antalis entalis*	150534	3	2	UC	Mollusca	Scaphopoda	Dentaliida	Dentaliidae
*Antalis vulgaris*	196380	3	2	UC/DC	Mollusca	Scaphopoda	Dentaliida	Dentaliidae
*Entalina tetragona*	139691	2	2	S	Mollusca	Scaphopoda	Gadilida	Entalinidae
Nematoda	799	2	2	S	Nematoda			
Cerebratulus	122348	4	3	B	Nemertea	Anopla		Cerebratulidae
Lineidae	122314	4	3	B	Nemertea	Anopla		Cerebratulidae
Nemertea	152391	4	3	B	Nemertea			
Tubularia	600947	1	1	E	Ochrophyta	Bacillariophyceae		
Phoronida	1789	2	1	S	Phoronida			Phoronidae
Phoronis	128545	2	1	S	Phoronida			
*Phoronis muelleri*	128549	2	1	S	Phoronida			
*Phoronis pallida*	128551	2	1	S	Phoronida			
Turbellaria	794	2	2	S	Platyhelminthes	Turbellaria		
Platyhelminthes	793	2	2	S	Platyhelminthes			
*Pachymatisma johnstonia*	134057	1	1	E	Porifera	Demospongiae	Astrophorida	Geodiidae
Golfingia	136021	4	3	B	Sipuncula	Sipunculidea	Golfingiida	Golfingiidae
*Golfingia (Golfingia) elongata*	175026	4	3	B	Sipuncula	Sipunculidea	Golfingiida	Golfingiidae
*Golfingia (Golfingia) iniqua*	136042	4	3	B	Sipuncula	Sipunculidea	Golfingiida	Golfingiidae
*Golfingia (Golfingia) vulgaris vulgaris*	410724	4	3	B	Sipuncula	Sipunculidea	Golfingiida	Golfingiidae
*Nephasoma (Nephasoma) minutum*	136060	4	3	B	Sipuncula	Sipunculidea	Golfingiida	Golfingiidae
*Thysanocardia procera*	136063	4	3	B	Sipuncula	Sipunculidea	Golfingiida	Golfingiidae
Onchnesoma	136024	4	3	B	Sipuncula	Sipunculidea	Golfingiida	Phascolionidae
*Onchnesoma magnibathum*	136064	4	3	B	Sipuncula	Sipunculidea	Golfingiida	Phascolionidae
*Onchnesoma steenstrupii steenstrupii*	410742	2	2	S	Sipuncula	Sipunculidea	Golfingiida	Phascolionidae
*Phascolion (Phascolion) strombus strombus*	410749	2	2	S	Sipuncula	Sipunculidea	Golfingiida	Phascolionidae
Sipunculidea	1296	4	3	B	Sipuncula	Sipunculidea		
*S*ipuncula	1268	4	3	B	Sipunculida			
*Aspidosiphon (Aspidosiphon) muelleri muelleri*	410717	4	3	B				
Ericthonius	101567	2	1	S				
*Goneplax rhomboides*	107292	4	4	B				
Isaeidae	101388	2	3	S				
Melitidae	101397	2	2	S				

M_*i*_ scores: 1 for organisms that live in fixed tubes; 2 indicates limited movement; 3 indicates slow, free movement through the sediment matrix; 4 indicates free movement, that is, via burrow system. R_*i*_ scores: 1 for epifauna; 2 for surficial modifiers; 3 for upward and downward conveyors; 4 for biodiffusors; and 5 for regenerators. Reworking types (Ft_*i*_): “S” for surficial modifiers; “B” for biodiffusors; “UC” and “DC” for upward and downward conveyors; and “R” for regenerators”.

## Discussion

As with any functional classification that on which BP_c_ is calculated relies on three main assumptions. Understanding these assumptions, and the need to correct for them where information is available, is key steps in the adequate use of BP_c_ as a metric:

1. If body size is constant, the BP_c_ of a species/taxon (BP_*i*_) is transferable across space and time. BP_c_ accounts for two “fixed” traits (R_*i*_ and M_*i*_) that are assumed to be directly related to life-history traits and activity levels of each species, that are not altered by context or spatiotemporal variation. Where information to the contrary is available about the alteration of species behavior in response to external stimuli, context-specific adjustments to reworking and mobility trait scores should be made accordingly: for example, thermal stress (Ouellette et al. 2004; Przeslawski et al. 2009); habitat structure (Godbold et al. 2011); ocean acidification (Godbold and Solan 2013); or presence of a predator (Maire et al. 2010). For instance, sediment type has been observed to be influential when determining the classification of a particular species into one of two specific functional groups (Needham et al. 2011), but this has not been documented for the vast majority of bioturbators. Incorporation of this type of information could be achieved using more sophisticated routines, such as fuzzy coding, to capture the influence of intraspecific variability in reworking and mobility traits (Maire et al. 2007; Bremner 2008; Godbold et al. 2011) across known sources of variation (habitat, season, food availability, etc.). The paucity of such information present for the majority of marine species (Tyler et al. 2012) is a source of concern and will be needed to project potential changes in BP_c_ under future policy or environmental scenarios. Typical body size is a “flexible” trait in the metric, which will vary in response to environmental variation, seasonality, stress, and disturbance (Queirós et al. 2006; Macdonald et al. 2012). BP_c_ captures this information through changes in the biomass/abundance ratio on which typical body size is calculated.
2. An organism's reworking (R_*i*_) and mobility (M_*i*_) modes remain the same across the life span of each individual. Thus far, variation in burrowing behavior across life stages has been poorly documented in the literature. However, in some species, juveniles and adults are known to exhibit different burial behavior, which can also be modified during reproductive stages (Aguzzi and Sardà 2007; Schwalb and Pusch 2009). If such changes in behavior are known to occur, different trait scores should be attributed as appropriate to different genders or life stages.
3. Where no species level information exists, taxa are assumed to have a similar bioturbation mode to others, which are closely related taxonomically. The paucity of information on many bioturbators (Teal et al. 2008) necessitates matching some species with the closest possible species or taxonomic group (e.g., genus or family). Regardless of whether taxonomic relatedness is a good indicator of the ecological characteristics of a species (Bevilacqua et al. 2012), the table presented here does not account for changes in taxonomic classification over time. Such changes in taxonomy could alter an organism's taxonomic relatedness to other taxa and therefore its assumed bioturbation classification. For example, *Alitta virens* (Sars 1835) and *Hediste diversicolor* (Müller 1776) may have been classified as being functionally similar, as both these species were formerly classified under the genus *Nereis* (Linnaeus 1758). Recent changes to their taxonomic classification now better reflect fundamental differences in bioturbation modes (i.e., gallery diffusor and conveyor, respectively, François et al. 2002). Nevertheless, in the absence of specific information, we consider it likely that genetically and physically similar taxa are likely to be functionally similar. As BP_c_ is a biomass-weighted sum of traits from many species, we consider it unlikely that small changes to trait assignments of individual species would greatly influence large-scale assessments. The current structure of the table reflects the taxonomic classification of the species at the time of analysis and will need updating as taxonomic inventories are refined.

Accepting the limitations imposed by the assumptions underpinning BP_c_, the classification list we have assembled will facilitate the calculation of BP_c_ across most temperate coast and shelf benthic environments in the North Atlantic. This aspects makes a strong case for a wider implementation of BP_c_ as a standardized indicator: the ability to build on existing data (abundance and biomass) to fill gaps about bioturbation patterns where direct assessments are not, or cannot, been routinely carried out. Further efforts will need to take place for other regions of the world or particular circumstances. There is also tremendous potential for application to historical datasets, to estimate bioturbation rates in the past, providing insight into how this process has helped to shape sedimentary ecosystems over time (Solan et al. 2008). Finally, the Marine Strategy Framework Directive (2008/56/EC) now requires an integrated understanding and management of regional scale patterns of marine ecosystem functioning, based on the use of inexpensive, rapid indicators. Few functional indicators currently exist for European waters, and initial tests of the suitability and applicability of BP_c_ suggest that it holds promise as a tool for informing management and policy (Van Hoey et al. 2010; Birchenough et al. 2012).
